# Supplementary material for: Dissecting transition cells from single-cell transcriptome data through multiscale stochastic dynamics
Source: Nat Commun. 2021 Sep 23;12:5609. doi: 10.1038/s41467-021-25548-w (PMC8460805; doi:10.1038/s41467-021-25548-w)
Supplement: Supplementary file 1 — Supplementary Information [file 41467_2021_25548_MOESM1_ESM.pdf]

Supplementary Material

Peijie Zhou[1,2], Shuxiong Wang[2], Tiejun Li[1,*], and Qing Nie[2,3,*]

[1]LMAM and School of Mathematical Sciences, Peking University, 100871 Beijing, China

[2]Department of Mathematics, University of California, Irvine, Irvine, CA 92697, USA

[3]Department of Cell and Developmental Biology, University of California, Irvine, Irvine, CA 92697, USA

[*]Correspondence: tieli@pku.edu.cn (T.L.), qnie@uci.edu (Q.N.)

Contents

Supplementary Note 1: Theoretical Analysis **3**

Supplementary Note 2: Algorithm Details **10**

Supplementary Note 3: Data Analysis Details **30**

Supplementary Note 4: Methods Comparison Details **57**

List of Figures

1 Dynamical system modeling of cell-fate transition and theoretical properties of MuTrans. 4
2 Framework of MuTrans. 11
3 Illustration of transition cell analysis. 24
4 MuTrans recovers dynamical structures of multi-stable potential wells . . 33
5 Dynamical system analysis of EMT data by MuTrans 35
6 Dynamical system analysis of early iPSC differentiation data by MuTrans. 38
7 MuTrans transition gene analysis from Pre-M to M states of iPSC data. . 40

8 Overall gene analysis toward the bifurcations into En/M states of iPSC data. 41
9 Dynamical system analysis of myelopoiesis data by MuTrans. 43
10 Dynamical manifold analysis of myelopoiesis data. 44
11 Transcendental results of myelopoiesis data for Multi-lin to Gran cell transitions. 45
12 Transcendental results of myelopoiesis data for HSPC to Meg cell transitions. 47
13 MuTrans results for the lymphoid lineage differentiation data. 50
14 MuTrans results for the human HSC differentiation data. 55
15 Comparison of MuTrans with other lineage inference methods in iPSC data. 60
16 Comparison of MuTrans with other lineage inference methods in myelopoiesis data. 63
17 Comparison of MuTrans transition cell score (TCS), Monocle 3 pseudotime, diffusion pseudotime(DPT), FateID fate bias probability and Palantir pseudotime to detect transition cells 65

List of Tables

1 The major adjustable parameters in MuTrans and Transcendental procedure. 27
2 Running time of MuTrans for different datasets on personal computers. . 28
3 Effect of changing first order tolerance on algorithm performance in iPSC dataset. 29
4 The list of MS, IH and TD genes in Pre-M to M transition of iPSC data 39
5 The list of MS, IH and TD genes in Multi-lineage to Gran transition of myelopoiesis data 46
6 The list of MS, IH and TD genes in HSPC to Meg transition of myelopoiesis data. 48
7 The list of MS, IH and TD genes in Pro-B to Pre-B transition of lymphoid lineage data. 53
8 Comparison of MuTrans with DPT and Palantir to calculate the pseudotime ordering in iPSC data. 66

Supplementary Note 1: Theoretical Analysis

Dynamical Systems Description of Cell-fate Transition

As a popular and basic description, the stochastic transition of cell-fate can be modeled by over-damped Langevin equation (OLE)

$$d\mathbf{X}_t = -\nabla U(\mathbf{X}_t)dt + \sqrt{2\varepsilon}d\mathbf{W}_t, \tag{1}$$

where $\mathbf{X}_t$ denotes the gene expression vector at time t, $\mathbf{W}_t$ denotes the standard Wiener process, accounting for the noise in gene expression dynamics and ε represents the noise amplitude of the system. Typically, the potential field $U(x)$ possesses several local minimals or potential wells, corresponding to the attractors of cell fate (Supplementary Fig. 1a).

Under small or moderate noise, the dynamical system will exhibit metastability property [1]. In most of the time, the gene expression of a single cell fluctuates gently around the attractors (Supplementary Fig. 1b). Driven by the noise or differentiation signal, switches between multiple macroscopic cell states may occur eventually [1, 2, 3, 4], creating the transition cells that are often traveling across the saddle point (also known as the transition state [5, 6] or intermediate state[7]) between attractor basins (Supplementary Fig. 1-ab).

Such metastability naturally introduces multi-scale model reduction (or coarse-graining) approaches to simplify the model computation and representation. When ε is small, the famous Kramers' reaction rate theory [8] establishes the quantitative relations between switch rate k_{ij} from attractor S_i to S_j with potential barrier height ΔU, stating that $k_{ij} \propto e^{-\Delta U/\varepsilon}$. Following rigorous analysis [9], the original OLE dynamics can then be upscaled as a Markov jump process, whose discrete states correspond to the attractors of OLE, and jump rate between states specified by k_{ij}.

It is thus interesting and important to investigate how the computational model of cell-fate transition can be combined with single-cell data analysis [10]. As shown in Supplementary Fig. 1-c, following Lafon and Coifman's results [11], the diffusion map random walk (DMRW) constructed on single-cell expression data [12, 13] yields the continuous limit which is exactly described by OLE dynamics. As a deeper exploration, below we will show in mathematically rigorous manner, that the coarse-grained Markov Chain of DMRW by MuTrans is consistent with the OLE model reduction by Kramers'

reaction rate theory, in the continuum limit and zero noise limit. As the result of such comprehensive understandings, various concepts in single-cell data analysis and computational modeling of cell-fate transition, can be therefore unified through MuTrans implementation (1d).

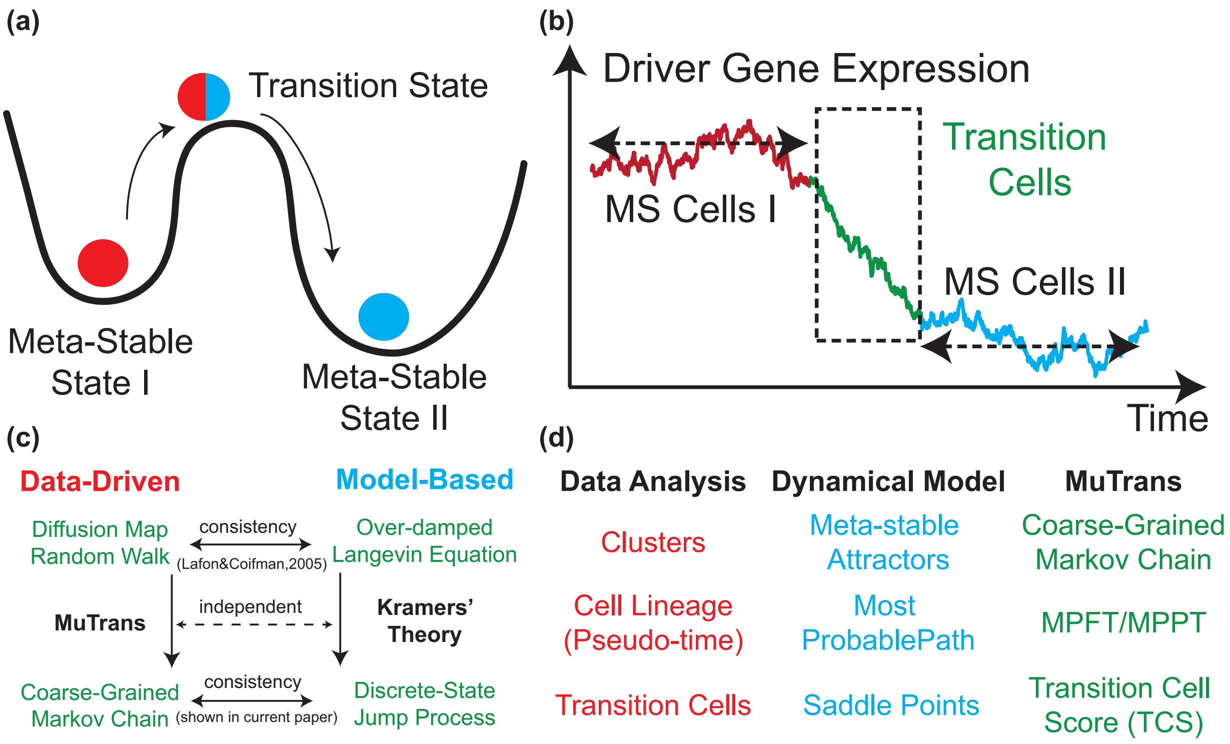

Supplementary Fig. 1: Dynamical system modeling of cell-fate transition and theoretical properties of MuTrans. (a) The notion of stable and transition cells modeled by double-well potential. (b) Typical gene expression dynamics of stable and transition cells in OLE model. (c) The theoretical consistency analysis of MuTrans. Despite the independence of methodology, the coarse-grained dynamics by MuTrans applied on Diffusion Map is mathematically equivalent to Kramers' reaction rate theory for OLE that serves for model reduction purposes. (d) The connections between data analysis and dynamical model concepts established by MuTrans.

Mathematical Analysis of MuTrans and OLE Consistency

To demonstrate the consistency, the key assumption on single-cell data is as follows, which is also the basis for the discussion by Lafon and Coifman about the continuum limit of Diffusion Map [11].

Assumption. *The single-cell data points* $\mathbf{x}_1, \mathbf{x}_2, ..., \mathbf{x}_n \in \mathbb{R}^d$ *are the i.i.d. samples from the distribution* $\rho(x)$*, with the Boltzmann-Gibbs form* $\rho(x) = e^{-\frac{U(x)}{\varepsilon}}$.

In MuTrans, the microscopic cell-cell dynamics $p(x, y)$ is constructed in the similar form with the diffusion map. The key procedure to construct the diffusion map is to define the weight function on the edge between data points x and y, in the scaled heat-kernel form,

$$w_{\delta,\alpha}(x, y) = \frac{k_\delta(x, y)}{q_\delta^\alpha(x) q_\delta^\alpha(y)}, \quad 0 \leq \alpha \leq 1.$$

where

$$k_\delta(x, y) = \frac{1}{z_\delta} e^{-\frac{\|x-y\|^2}{4\delta}}, \quad z_\delta = (4\pi\delta)^{d/2} \tag{2}$$

and $q_\delta(x) = \sum_{y \in S} k_\delta(x, y)$, here S denotes the whole sets of data points. The transition probability of diffusion-map random walk is therefore defined as

$$p_{\delta,\alpha}(x, y) = \frac{w_{\delta,\alpha}(x, y)}{d_{\delta,\alpha}(x)},$$

where $d_{\delta,\alpha}(x) = \sum_{y \in S} w_{\delta,\alpha}(x, y)$. The backward operator of the process can also be defined as $\mathcal{T}_b^{\delta,\alpha}\phi(x) \triangleq \sum_y p_{\delta,\alpha}(x, y)\phi(y)$.

The continuum limit of diffusion map process mainly involves two limits, the large-sample limit $n \to \infty$ in the first step and the small band-width limit $\delta \to 0$ in the second step.

To find the limit of $n \to \infty$, the central technique is the law of large numbers, stating that $\frac{1}{n}\sum_{i=1}^n f(x_i) \to \int f(x)\rho(x)dx$ under appropriate conditions for the measurable function f and the random variable. Applying to the expression of $\mathcal{T}_b^{\delta,\alpha}\phi(x)$, we have

$$\lim_{n\to\infty} \mathcal{T}_b^{\delta,\alpha}\phi(x) = \frac{\int \phi(y)\tilde{q}_\delta^{-\alpha}(y) k_\delta(x, y)\rho(y)dy}{\int \tilde{q}_\delta^{-\alpha}(y) k_\delta(x, y)\rho(y)dy}, \tag{3}$$

where

$$\tilde{q}_\delta = \int k_\delta(x, y)\rho(y)dy.$$

To find the limit of $\delta \to 0$, the following lemma is important, which can also be obtained from Laplace integral formula.

Lemma 1. *For the smooth function $\phi(x)$, we have*

$$\int_{R^d} k_\delta(x,y)\phi(y) = \phi(x) + \delta(\Delta\phi(x) + h(x)\phi(x)) + O(\delta^{3/2}),$$

where $h(x)$ is independent of $\phi(x)$.

Applying the lemma to Equation (3) and collecting the leading orders, we find the $O(1)$ term is $\phi(x)$, and the $O(\delta)$ term $\frac{\Delta(\phi\rho^{1-\alpha})}{\rho^{1-\alpha}} - \phi\frac{\Delta(\rho^{1-\alpha})}{\rho^{1-\alpha}}$. When $\alpha = 0.5$, we arrive at the famous conclusion by Lafon and Coifman [11], connecting the dynamics of diffusion-map random walk and over-damped Langevin equaion,

Theorem 2. *[11]*

$$\lim_{\delta\to 0}\lim_{n\to\infty} \varepsilon\delta^{-1}(\mathcal{T}_b^{\delta,1/2} - I)\phi(x) = -\nabla U \cdot \nabla\phi + \varepsilon\Delta\phi,$$

where the limiting operator correspond to the infinitesimal generator of SDE

$$d\mathbf{X}_t = -\nabla U(\mathbf{X}_t)dt + \sqrt{2\varepsilon}d\mathbf{W}_t, \tag{4}$$

whose invariant distribution is exactly $\rho(x)$.

The theorem suggests if the data-points are sampled from the invariant distribution of SDE (4), then we can recover the original dynamics directly from data when inspecting the random walk defined by diffusion maps with time step $O(\delta)$. This result coincides with the intuitions to construct Brownian motion by invariant principle [14], since the space correlation scale from Gaussian kernel $k_\delta(x,y)$ is $O(\sqrt{\delta})$, matching with time scale of $O(\delta)$. From this perspective, the microscopic $p(x,y)$ constructed in our single-cell algorithm reflects the over-damped Langevin dynamics underlying the cell-fate decision process.

Next we move further by asking the following question. As discussed, MuTrans can provide with a natural way for the multi-scale reduction of diffusion map random walk. On the other hand, the classical way to directly coarse-grain the OLE dynamics (4) is from Kramers reaction rate formula, by viewing the coarse-grained dynamics as the discrete-state jump process, with transition rate which directly relates to the barrier height of $U(x)$. Are the two coarse-graining ways equivalent, if the data is indeed sampled from the stationary distribution of SDE?

Using MuTrans to study diffusion map dynamics $p^{\delta,\alpha}$, we can calculate the coarse-grained transition probability matrix $\hat{\mathbf{P}}^{\delta,\alpha}$, where the transition probability from S_1 to

S_2 is

$$\hat{P}_{12}^{\delta,\alpha} = \sum_{x \in S_1, y \in S_2} \mu_1^{\delta,\alpha}(x) p_{\delta,\alpha}(x,y), \quad \mu_1^{\delta,\alpha}(x) = \frac{d_{\delta,\alpha}(x)}{\sum_{x \in S_1} d_{\delta,\alpha}(x)}.$$

For simplicity of discussion, below we restrict to the one dimensional case, and assume $U(x)$ is the double-well potential with two local minimum at $x = a$ and $x = b$, plus the saddle point at $x = c$. We also assume that the stable states S_1 and S_2 are accurate in MuTrans. Then as the sample number tends to infinity, we have

$$\lim_{n \to \infty} \hat{P}_{12}^{\delta,\alpha} = \frac{\iint_{(-\infty,c] \times [c,+\infty)} \tilde{q}_\delta^{-\alpha}(x) \tilde{q}_\delta^{-\alpha}(y) k_\delta(x,y) \rho(x) \rho(y) dx dy}{\int_{-\infty}^{c} \tilde{d}^{\delta,\alpha}(x) \rho(x) dx},$$

where

$$\tilde{d}^{\delta,\alpha}(x) = \tilde{q}_\delta^{-\alpha}(x) \int_R \tilde{q}_\delta^{-\alpha}(y) k_\delta(x,y) \rho(y) dy.$$

To find the limit as $\delta \to 0$, we need the following lemma.

Lemma 3. *For the smooth function $f(x,y)$ on $\mathbb{R}^2$, we have*

$$\sqrt{\frac{\pi}{\delta}} \iint_{(-\infty,c] \times [c,+\infty)} k_\delta(x,y) f(x,y) dx dy = f(c,c) + O(\delta).$$

This can be shown by considering the Laplace method in $\mathbb{R}^2$, since in the integral region $(-\infty, c] \times [c, +\infty)$ only the point (c, c) contributes to the weight in $k_\delta(x, y)$. Therefore, apply the Taylor's expansion to $f(x, y)$ around (c, c), and note that the integral

$$\iint_{(-\infty,c] \times [c,+\infty)} e^{-(4\delta)^{-1}(x-y)^2} dx dy = \frac{1}{2} \iint_{\Omega_{u,v}} e^{-(4\delta)^{-1}u^2} du dv = 2\delta,$$

where $\Omega_{u,v} = \{(u, v) | u + v \leq 0, v - u \geq 0\}$, through the change of variable $u = x - y$ and $v = x + y$. Combined with (2), we find the leading order of $O(\sqrt{\delta})$ in the integral of lemma 3. Hence, we conclude that

$$\lim_{\delta \to 0} \sqrt{\frac{\pi}{\delta}} \hat{P}_{12}^{\delta,\alpha} = \frac{\rho^{2-2\alpha}(c)}{\int_{-\infty}^{c} \rho^{2-2\alpha}(x) dx}. \tag{5}$$

Since we consider the state-transition under small noise, after taking the $\delta \to 0$ limit, we are also interested limit regarding small noise $\varepsilon \to 0$. Applying the Laplace integral asymptotic for $\rho(x) = e^{-\varepsilon^{-1} U(x)}$, and pluging in Equation (5), we finally arrive at

Theorem 4.

$$\lim_{\varepsilon \to 0} \lim_{\delta \to 0} \lim_{n \to \infty} \varepsilon \ln \delta^{-1/2} \hat{P}_{12}^{\delta,\frac{1}{2}} = U(a) - U(c).$$

The theorem implies the consistency between MuTrans-based coarse-graining and the Kramers reaction rate formula for OLE. Consider a continuous-time, two-state jump process with generator $\mathbf{Q}$. Then if we sample the trajectory with fixed time increment Δt, a discrete-time Markov chain can be induced, with transition probability matrix (TPM) $\mathbf{P}(\Delta t) = \exp(\Delta t \mathbf{Q})$. Conversely, we have $\mathbf{Q} = \lim_{\Delta t \to 0} \frac{\mathbf{P}(\Delta t) - I}{\Delta t}$, which is the original definition of infinitesimal generator. Then the results in Theorem 4 suggest that when resolving the coarse-grained TPM with time scale $O(\sqrt{\delta})$, the limiting jump process asymptotically correspond to the reduced state-transition process directly from the original SDE by Kramers reaction rate, as the noise of the system $\varepsilon \to 0$.

Through the theoretical analysis above, we therefore conclude that MuTrans can reflect the multi-level dynamics of the single-cell transcriptome data modeled by the over-damped Langevin equation, in a quantitatively consistent way.

Further Discussions

Presently, the interplay between single-cell data analysis and dynamical models in computational biology can be realized in two diverging ways, namely the model-based approach and data-based approach respectively.

The model-based approach assumes the existence of a generative model (commonly a *continuous* dynamical systems) where the single-cell data is sampled from, and analyze the gene expression dynamics via fitting dozens of parameters in computational model. For example, by modeling the gene regulation network with ordinary differential equation (ODE), Ocone et al. [15] proposed a framework to reconstruct the gene regulatory dynamics from single-cell snapshot data. Subsequently, SCUBA [16] involved the fitting of a bifurcating ODE system to describe the gene expression dynamics in time-series data. SCOUP [17] inferred the cell lineage and pseudotime via the parameter estimation for the conceived Ornstein-Uhlenbeck process, which was a special class of stochastic differential equation (SDE). The framework had been extended to time-series data with the consideration of more general Gaussian process dynamics [18, 19, 20]. Recently, partial differential equation (PDE) models were also adopted to simulate the pseudo-dynamics (population density evolution) in the single-cell RNAseq time series [21].

On the other hand, data-based approach directly defines stochastic dynamics on data points, yielding *discrete* models in both space and time. For instance, diffusion map and diffusion pseudotime [13] were among the earliest proposals to construct stochastic dynamics directly on single-cell data. The population balance analysis (PBA) method

[22] also utilized discrete random walk on the cellular graph to infer cell state dynamics, and discussed in detail the relationship between the constructed random walks and the continuous partial differential equation. In addition, the Topographer [23] and Palantir [24] also applied the discrete Markov chain model to gain a probabilistic understanding of the cell fate decision.

In general, the model-based methods provide more mechanism understandings, while tend to be problem-restrictive and computation-expensive; on the contrary, the data-based methods usually provide more robust and scalable results, comprising with the sacrifice of model interpretability.

MuTrans combines the advantages of both approaches by constructing a data-based cell-cell random walk in the first step, and interpreting and decomposing the dynamics from a multi-scale stochastic model perspective, to perform cell clustering, lineage inference and transition cells/genes analysis in a consistent and mathematically rigorous way.

Supplementary Note 2: Algorithm Details

As an appendix to main text, in this part we present the MuTrans along with downstream Transcendental algorithm and the large-scale dataset pre-processing module DECLARE with more technical details, with the framework outlined in Supplementary Fig. 2.

The central task of MuTrans is to extract three key dynamical information from single-cell transcriptome data, namely

- The attractor basins $S = \bigcup_{k=1}^{K} S_k$,
- The mutual transition probabilities among attractor basins $\hat{\mathbf{P}}_{K\times K}$,
- The relative cell positions within attractors (membership function) $\rho_k(x)$.

To this end, we use $\{S_k, \hat{P}_{ij}, \rho_k(x)\}$ to induce two random walk transition probability matrix (rwTPM) on

- cluster-cluster resolution, $\hat{p}(x, y)$, which relies on S_k and $\hat{P}_{ij}$,
- cell-cluster resolution, $\tilde{p}(x, y)$, which relies on $\hat{P}_{ij}$ and $\rho_k(x)$.

Then we optimize $\{S_k, \hat{P}_{ij}, \rho_k(x)\}$ to ensure that the two model-based rwTPM $\hat{p}$ and $\tilde{p}$ best approximate the cell-cell resolution rwTPM p, which is directly constructed from single-cell data via a diffusion-like kernel.

Finally, the downstream analysis (Transcendental) is performed on the obtained optimal $\{S_k^*, \hat{P}_{ij}^*, \rho_k^*(x)\}$ to visualize the cell-fate dynamical manifold, infer the cell lineage and dissect transition cells and relevant genes.

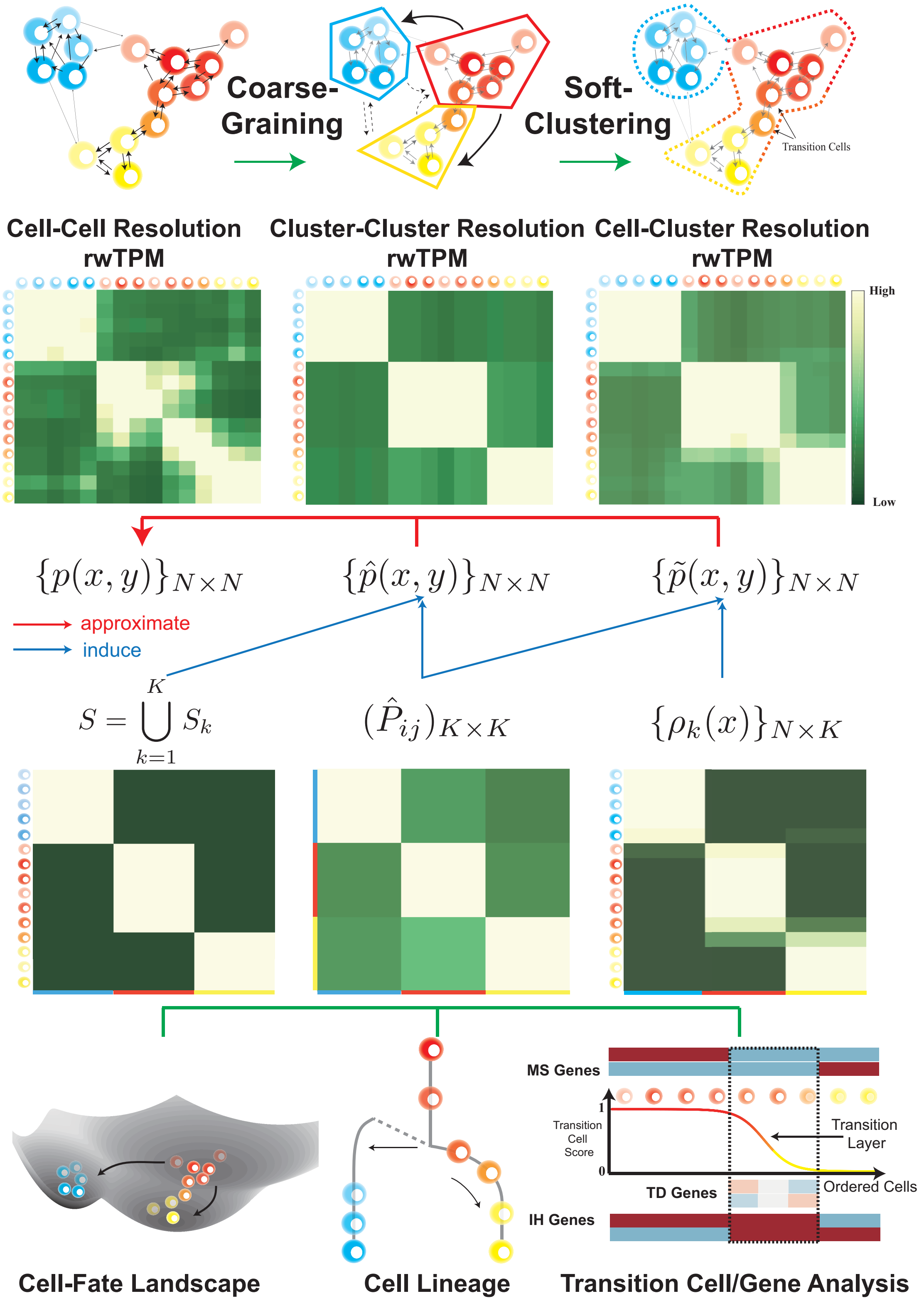

Supplementary Fig. 2: Framework of MuTrans. By unifying three rwTPM $p, \hat{p}$ and $\tilde{p}$ across cell-cell, cluster-cluster and cell-cluster resolutions, MuTrans extracts a coarse-grained Markov Chain representing original dynamics, with attractor basins S_k and mutual conversion probability $\hat{P}$, and specifies the relative positions of single cells within attractor basins via membership function $\rho_k(x)$. The extracted quantities are then used in downstream analysis such as dynamical manifold construction, cell lineage inference or transition cells and genes analysis.

Construction of Microscopic Cellular Random Walk

We begin to construct the random walk on the cell-cell level by defining a similarity function $g(x, y)$ in Gaussian kernel form, expressed as $g(x, y) = e^{-\frac{m^2(x,y)}{\sigma^2(x)}}$, where $m(x, y)$ is the metric between cell x and y, and $\sigma(x)$ is the local standard deviation of the Gaussian kernel concentrated around cell. In our algorithm, the choice of metric m can be either Euclidean, correlation or cosine, and the value of σ can be obtained by the perplexity parameter as in tSNE. Based on the Gaussian kernel similarity, we then introduce the weight function $w(x, y)$ between any pair of cells by making $g(x, y)$ symmetric, i.e, $w(x, y) = \frac{1}{2}(g(x, y) + g(y, x))$. The weighted random walk with $w(x, y)$ can hence be modeled as the Markov chain with transition probability $p(x, y)$ such that

$$p(x, y) = \frac{w(x, y)}{d(x)}, \quad d(x) = \sum_z w(x, z).$$

Here $p(x, y)$ denotes the probability that the cell currently in state x will switch to the state y in one step of the random walk. Such microscopic random walk yields an equilibrium probability distribution $\mu(x) = \frac{d(x)}{\sum_z d(z)}$, which satisfies the detailed balance condition that $\mu(x)p(x, y) = \mu(y)p(y, x)$. Under the random walk perspective of single-cell data, the dynamics of gene expression can be viewed as a series of consecutive stochastic switches from one cell to another, which is also consistent with the OLE dynamics (1).

Multi-scale Representation of Stochastic Dynamics

The aim of the multi-scale representation algorithm is to dissect the original, microscopic random walk dynamics $\{p(x, y), x, y \in S\}$ into the multi-scale structure

$$\left(\{S_k\}_{k=1}^K, \{\hat{P}_{ij}\}_{i,j=1}^K, \{\rho_k(x), x \in S\}_{k=1}^K \right).$$

Here $\{\hat{P}_{ij}\}_{i,j=1}$ defines a coarse-grained Markov chain on the states $\{S_k\}_{k=1}^K$, which is the partition of the microscopic space S and corresponds to the concept of attractor basins of dynamical system. The $\{\rho_k(x), x \in S\}_{k=1}^K$ specifies the probability of cell x belongs to the attractor basin S_k, determining the relative position of the cells in the holistic landscape. The underlying multi-scale structure naturally induces the dynamics on microscopic cellular level as defined below.

The Cluster-Cluster Resolution Dynamics. The coarse-grained Markov chain

with the structure $\left(\{S_k\}_{k=1}^K, \{\hat{P}_{ij}\}_{i,j=1}^K\right)$ naturally induces a dynamics $\hat{p}[S_k, \hat{P}_{ij}]$,

$$\hat{p}(x,y) = \sum_{i,j=1}^{K} 1_{S_i}(x)1_{S_j}(y)\hat{P}_{ij}\frac{\mu(y)}{\hat{\mu}_j}, \quad \hat{\mu}_j = \sum_{y\in S_j}\mu(y). \tag{6}$$

Here $1_{S_k}(z)$ denotes the indicator function of cluster S_k such that $1_{S_k}(z) = 1$ for cell $z \in S_k$ and $1_{S_k}(z) = 0$ otherwise. It is worth noting that, although $\hat{p}(x,y)$ is defined at the cell-cell level, it is indeed induced from the transitions in cluster-cluster resolution: from the cluster-cluster transition perspective, the stochastic transition from cell $x \in S_i$ to $y \in S_j$ can be decomposed into a two-stage process; the cell witnesses the switch of cellular state from cluster S_i to S_j with probability $\hat{P}_{ij}$ in the first stage, and then pick up the cell y in cluster S_j according to its relative portion at equilibrium $\frac{\mu(y)}{\hat{\mu}_j}$.

The Cell-Cluster Resolution Dynamics. Certain dynamics information might be lost during the coarse-graining, especially regarding the saddle points that represent the transition cells amid attractor basins, since the cluster-cluster resolution dynamics in Equation (6) assigns these cells to one specific cluster exclusively. In order to discover the transition cells in higher resolution, we introduce the membership function $\{\rho_k(x), x \in S\}_{k=1}^K$ to quantify the relative cell positions in the clusters. The element $\rho_k(x)$ represents the probability that the cell x belongs to cluster S_k and we require that $\rho_k(x) \geq 0, \sum_k \rho_k(x) = 1$ for any $x \in S$. If the gene expression profile of the cell z is distinctive to the cluster S_k, then $\rho_k(z)$ will approach to one as the indicator function 1_{S_k}. On the other hand, for the cell possessing mixed lineage identities, its membership function $\rho_k(z)$ will have several significant positive components, suggesting its potential origin and destination during the transition process. The idea of membership function is sometimes referred to "soft clustering" previously [25], since the boundary among the discrete clusters are softened by assigning cells the probability belonging to certain clusters, which takes the value continuously from 0 to 1. With the introduction of $\{\rho_k(x), x \in S\}_{k=1}^K$, we are able to induce a refined microscopic dynamics $\tilde{p}[\hat{P}_{ij}, \rho_k(x)]$ beyond Equation (6) as

$$\tilde{p}(x,y) = \sum_{i,j=1}^{K} \rho_i(x)\rho_j(y)\hat{P}_{ij}\frac{\mu(y)}{\hat{\mu}_j}, \quad \hat{\mu}_j = \sum_{y\in S}\rho_j(y)\mu(y). \tag{7}$$

In the current set-up, since the membership of the cell to cluster is no longer exclusive, the transition from cell x to y can be realized in all the possible channels from any cluster S_i to S_j with the weight $\rho_i(x)\rho_j(y)$. Therefore, the dynamics in Equation (7) recovers

information from the cell-cluster resolution.

The Optimal Multi-scale Representation. We aim to find the multi-scale representation $\Big(\{S_k\}_{k=1}^K, \{\hat{P}_{ij}\}_{i,j=1}^K, \{\rho_k(x), x \in S\}_{k=1}^K\Big)$, whose induced random walks $\hat{\mathbf{p}}$ and $\tilde{\mathbf{p}}$ optimally approximate the original data-driven dynamics $\mathbf{p}$. As pointed out in [26], the natural choice to measure the distance between Markov chain dynamics is the Hilbert-Schmidt norm for the operator, defined as $\|\mathbf{p}\|_\mu^2 = \sum_{x,y\in S} \frac{\mu(x)}{\mu(y)} p^2(x,y)$, where μ is the invariant distribution of $\mathbf{p}$. Therefore, we first formulate following the optimization problem to detect the optimal coarse-grained Markov-chain $\Big(\{S_k\}_{k=1}^K, \{\hat{P}_{ij}\}_{i,j=1}^K\Big)$,

$$\begin{aligned} \min_{\{S_k\}_{k=1}^K, \{\hat{P}_{ij}\}_{i,j=1}^K} & \mathcal{E}_1[S_k, \hat{P}_{ij}] = \|\hat{\mathbf{p}}[S_k, \hat{P}_{ij}] - \mathbf{p}\|_\mu^2, \\ \textbf{s.t.} \quad & \bigcup_{k=1}^K S_k = S, \quad S_i \cap S_j = \varnothing, \quad \text{for } i,j \in \{1,2,..,K\}, \\ & \hat{P}_{ij} \geq 0, \quad \sum_{j=1}^K \hat{P}_{ij} = 1, \quad \text{for } i,j \in \{1,2,..,K\}. \end{aligned} \tag{8}$$

The solved optimal $\Big(\{S_k^*\}_{k=1}^K, \{\hat{P}_{ij}^*\}_{i,j=1}^K\Big)$ further facilitates the determination of $\{\rho_k(x)\}_{k=1}^K$ through

$$\min_{\{\rho_k(x), x\in S\}_{k=1}^K} \mathcal{E}_2[\rho_k(x)] = \|\tilde{p}[\hat{\mathbf{P}}_{ij}^*, \rho_k(x)] - \mathbf{p}\|_\mu^2, \tag{9}$$

$$\textbf{s.t.} \quad \rho_k(x) \geq 0, \quad \sum_{k=1}^K \rho_k(x) = 1, \quad \text{for } k \in \{1,2,..,K\}, x \in S, \tag{10}$$

with the initial condition $\rho_k^0(x) = 1_{S_k^*}(x)$ for any $x \in S$.

The Coarse Graining Procedure. We aim to solve the minimization problem by the iteration scheme,

$$\begin{aligned} \hat{P}_{ij}^{(t+1)} &= \operatorname*{argmin}_{\{\hat{P}_{ij}\}_{i,j=1}^K} \mathcal{E}_1[S_k^{(t)}, \hat{P}_{ij}], \\ S_k^{(t+1)} &= \operatorname*{argmin}_{\{S_k\}_{k=1}^K} \mathcal{E}_1[S_k, \hat{P}_{ij}^{(t)}]. \end{aligned} \tag{11}$$

We expand the objective function in optimization problem (8) as

$$\mathcal{E}_1[S_k, \hat{P}_{ij}] = \sum_{x,y\in S} \frac{\mu(x)}{\mu(y)} p^2(x,y) + \sum_{i,j=1}^K \frac{\hat{\mu}_i}{\hat{\mu}_j} \hat{P}_{ij}^2 - 2 \sum_{i,j=1}^K \frac{1}{\hat{\mu}_j} \hat{P}_{ij} \sum_{x\in S_i, y\in S_j} \mu(x) p(x,y), \tag{12}$$

Since the objective function yields the quadratic form with respect to $\{\hat{P}_{ij}\}_{i,j=1}^K$, the problem can be tackled explicitly

$$\hat{P}_{ij}^{(t+1)} = \sum_{x\in S_i^{(t)}, y\in S_j^{(t)}} \mu_i^{(t)}(x)p(x,y), \quad \mu_i^{(t)}(x) = \frac{\mu(x)}{\hat{\mu}_i^{(t)}}, \quad \hat{\mu}_i^{(t)} = \sum_{y\in S_i^{(t)}} \mu(y). \tag{13}$$

We can verify that the obtained $\hat{P}_{ij}^{(t+1)}$ satisfy the constrains in problem (8). The original problem turns out to be the NP-hard combinatorial optimization. To overcome such limitation, a heuristic algorithm has been proposed [26] that shared the spirit with K-means clustering. To present the heuristic method, we rewrite the objective function as

$$\mathcal{E}_1[S_k, \hat{P}_{ij}] = \sum_{i=1}^K \sum_{x\in S_i} \bar{\mathcal{E}}_i(x; S_k, \hat{P}_{ij})$$

where

$$\bar{\mathcal{E}}_i(x; S_k, \hat{P}_{ij}) = \sum_{j=1}^K \sum_{y\in S_j} \mu(x)\mu(y) \left(\frac{p(x,y)}{\mu(y)} - \frac{\hat{P}_{ij}}{\hat{\mu}_j}\right)^2$$

The update of S_k can be achieved by the greedy step

$$S_k^{(t+1)} = \{x : k = \operatorname*{argmin}_i \mathcal{E}_i(x; S_k^{(t)}, \hat{P}_{ij}^{(t)})\}, \tag{14}$$

where

$$\mathcal{E}_i(x; S_k^{(t)}, \hat{P}_{ij}^{(t)}) = \sum_{j=1}^K \sum_{y\in S_j^{(t)}} \mu(x)\mu(y) \left(\frac{p(x,y)}{\mu(y)} - \frac{\hat{P}_{ij}^{(t)}}{\hat{\mu}_j^{(t)}}\right)^2.$$

The Refinement Procedure. Given the coarse-grained results $\{S_k^*\}_{k=1}^K$ and $\{\hat{P}_{ij}^*\}_{i,j=1}^K$, the main obstacle of solving the optimal refinement representation problem (9) arises from the constraints for $\rho_k(x)$. To overcome the difficulty, we introduce the change of variable $\rho_k(x) = e^{\lambda_k(x)}/\sum_{k=1}^K e^{\lambda_k(x)}$. Here the transformed variable $\{\lambda_k(x)\}_{k=1}^K \in \mathbb{R}$ guarantees the constraints that $\rho_k(x) \geq 0, \sum_{k=1}^K \rho_k(x) = 1$, hence they do not subject to any further constraints. If we define $\tilde{\mathcal{E}}_2[\lambda_k(x)] = \mathcal{E}_2[\rho_k(x)]$, then the refinement problem (9) can be tackled via the classical quasi-Newton method with respect to objective function $\tilde{\mathcal{E}}_2[\lambda_k(x)]$ and the variables $\{\lambda_k(x)\}_{k=1}^K$.

Construction of Dynamical Manifold

To construct the dynamical manifold in single-cell analysis, challenges arise from the curse of dimensionality. For the high dimensional transcriptome dataset, the appropriate

illustration of dynamical manifold depends on the reasonable low dimensional embedding of the cells that represents the ordering of cell-fate transition dynamics. To solve the issue, we define a transition coordinate $\xi(x)$ on the two-dimension plane, based on the multi-scale dynamics property.

Our dimension reduction procedure involves two steps. In the initial step, we determine the center positions of each cell clusters, which correspond to the attractors of corresponding dynamical systems. In the second step, we assign the coordinate to each individual cell by considering the transition cell information. The initial center-determination step starts with an appropriate two-dimensional representation $\mathbf{x}^{2D}$ of each cell x, which can be realized by the spectral visualization of stochastic dynamics combined with MDS techniques, or simply by classical method like Diffusion Maps or tSNE. Instead of directly utilizing $\mathbf{x}^{2D}$ as the cell coordinate in landscape function, we calculate the center $\mathbf{Y}_k$ of cluster S_k^*, by taking the average coordinate over cells within certain threshold of cluster membership function $\rho_k^*(x)$, i.e.

$$\mathbf{Y}_k = \frac{\sum\limits_{x\in S} \mathbf{x}^{2D} 1_{\rho_k^*(x)>h_{2D}}(x)}{\sum\limits_{x\in S} 1_{\rho_k^*(x)>h_{2D}}(x)}, \quad k \in \{1, 2, \ldots, K\}.$$

The threshold h_{2D} allow us not only to consider the cells that tightly attracted to the fixed point in attractor basin S_k^*, but also to include cells that are in the transition process from or toward the attractor basin. Having determined the position of attractors, we define the two-dimensional transition coordinate $\xi(x)$ according to the membership function $\rho_k^*(x)$ in different attractor basins, such that

$$\xi(x) = \sum_{k=1}^{K} \rho_k^*(x) \mathbf{Y}_k \in \mathbb{R}^2. \tag{15}$$

For the cell possessing mixed identities of state S_i^* and S_j^*, its transition coordinate will lie in the middle of y_i and y_j. In each attractor basin S_k^* , we can also obtain the local covariance matrix Λ_k of the transition coordinate $\xi(x)$, i.e.

$$\Lambda_k = \mathrm{Cov}[\xi(x)|\rho_k^*(x) > h_{2D}]$$

which represents the distribution of cells relevant to the basin. Next, we aim to define the landscape function that describes the global stochastic transitions among the multiple attractors in the system. We piece the local information of different attractor basins

together by fitting a Gaussian mixture model with mixture weight given by $\hat{\mu}^*$, the stationary distribution of coarse-grained dynamics. The probability distribution function $\mathcal{P}(z)$ of the mixture model can be written as

$$\mathcal{P}(z) = \sum_{k=1}^{K} \hat{\mu}_k^* \mathcal{N}(z; \mathbf{Y}_k, \Lambda_k),$$

where $\mathcal{N}(z; \mathbf{Y}_k, \Lambda_k)$ is the 2-dimension Gaussian probability density function with mean y_k and covariance Λ_k. The landscape function ϕ is defined on the 2-dimension space for any point z such that $\phi(z) = -\ln \mathcal{P}(z)$ and visualized as the dynamical manifold. Specifically, the function of individual cell x is calculated as $\phi(\xi(x))$.

We argue that the landscape function ϕ and its associated dynamical manifold accommodate rich information about the multi-scale stochastic dynamics during cell fate decisions. Firstly, the re-arranged transition coordinates $\xi(x)$ allow typical cells that are distinctive to certain cell states are positioned in the basin around corresponding attractors $\{\mathbf{Y}_k\}_{k=1}^K$, while the transition cells are laid along the connecting path between attractors across the saddle point, according to membership function $\rho_k^*(x)$. Secondly, the relative depth of the attractor basin reflect the stationary distribution $\hat{\mu}^*$ of coarse-grained dynamics $\left(\{S_k^*\}_{k=1}^K, \{\hat{P}_{ij}^*\}_{i,j=1}^K\right)$, therefore depicting the relative stability of the cell state in the stochastic transition dynamics among different states. Moreover, the flatness of the attractor basin is determined by the local covariance matrix Λ_k, which reveals the abundance and distribution of transition cells, and therefore the sharpness of cell fate switch.

Compared with the existing landscape proposals, distinctive features about our method arise in two aspects. Firstly, we resolved the curse of dimensionality issue in landscape visualization by the introduction of transition coordinates, which incorporated multi-scale dynamics information. In previous attempts, in order to visualize certain energy landscape in three-dimension space, the cells were projected onto the two-dimension plane by applying classical dimension reduction methods like PCA, tSNE or elastic embedding, which might lack dynamical interpretations. As the improvement, the transition coordinates in our method faithfully reflect the progress of cell development, by assigning and sorting the transition cells along the connecting path between their departing and targeting stable states. Secondly, the dynamical manifold in MuTrans purely serves as the descriptive tool to vividly and accurately demonstrate the dynamical information about cell fate conversion process. Some previous proposals further utilized the exact

value of energy landscape to infer the underpinning dynamics, which introduced further assumptions about the dynamical models. For instance, scEpath [27] inferred the cluster-cluster transition probability by assuming that the equilibrium distribution yielded the Boltzmann-Gibbs form with the constructed signaling-entropy energy landscape. Meanwhile, DensityPath [28] probed the development trajectory with the hypothesis that the least action path corresponded to the geodesic curve on the density landscape. In comparison, our multi-scale representation of cell state transition dynamics and the downstream analysis were all directly and optimally derived from of the original high-dimension space of gene expression, independent of the visualized dynamical manifold. Consequently, the inference results toward the transition process by our method might be applicable to more general cases, and the relevant demonstration-aimed dynamical manifold seems to be closer to the original idea in Waddington's metaphor.

The Transcendental Procedure

Following the results of MuTrans, the Transition Cell and Relevant Analysis (Transcendental) procedure performs three tasks relevant to the transition cells

- Inferring the cell lineage (transition trajectory) on cell states level;
- Identifying the transition cells with the defined transition cell score (TCS);
- Distinguishing the transition driver (TD), intermediate-hybrid (IH) or meta-stable (MS) genes during interested transition process.

Inference of the Cell Lineage.

Beyond the visualization of dynamical manifold, MuTrans also provides more quantitative approaches to infer the cell lineage from the transition dynamics. Based on the MuTrans coarse-grained reduction $\left(\{S_k^*\}_{k=1}^K, \{\hat{P}_{ij}^*\}_{i,j=1}^K\right)$, a cluster-cluster graph $\mathcal{G}(V, E, W)$ naturally emerges, whose nodes $\{V_k\}_{k=1}^K$ denote the coarse-grained cell states $\{S_k^*\}_{k=1}^K$, and nonnegative weights W_{ij} on edge E_{ij} can be computed from the cell state transition probability $\hat{P}_{ij}^*$ (described below).Given the initial attractor, a tree-like structure is then subtracted from $\mathcal{G}(V, E, W)$ to represent the cell lineage during development. Depending on the different choice of weight matrix and tree construction algorithm, Transcendental provides the following two methods to infer cell lineage, namely the Maximum Probability Flow Tree (MPFT) and Most Probable Path Tree (MPPT) approaches.

The Maximum Probability Flow Tree (MPFT). The coarse-grained Markov chain on cluster-cluster level satisfies the detailed balance condition, i.e. $\hat{\mu}_i^* \hat{P}_{ij}^* = \hat{\mu}_j^* \hat{P}_{ji}^*$, therefore introducing a symmetric probability flow matrix F defined by $F_{ij} = \hat{\mu}_i^* \hat{P}_{ij}^*$. Here flow F_{ij} denotes the percentage of cells that are making transitions from state S_i^* to S_j^*, against all the cells in transition during one-step evolution of coarse-grained Markov chain. We aim to build a maximum spanning tree of the undirected graph with weights $W = F$, such that the tree possesses the greatest sum of transition probability flows over all pair of edges, which represents the dominating transition pathways of the coarse-grained dynamics. In Transcendental implementation, the resulting Maximum Probability Flow Tree (MPFT) is constructed by employing the Kruskal's algorithm on weight matrix F.

The Most Probable Path Tree (MPPT) Unlike MPFT which seeks to probe a global tree structure that maximize the total transition flux, Transcendental also provides the Most Probable Path Tree (MPPT) to represent the transition paths from root state to every cell state, which is based on the transition path theory [29, 30, 31, 32, 33, 34].

Given the sets of root and ending clusters, denote them as starting set A and the target set B, respectively. Suppose we already derive the coarse-grained cluster-cluster scale Markov chain X_t with transition probability $\hat{\mathbf{P}}$.

Below we first define the core concepts in transition path theory as follows which can be found in previous literatures.

Definition 1 (In-Transition Times [34]). *For a given path $\{X_t\}$, the in-transition times from set A to B are defined as the union of sets*

$$\mathscr{T} = \bigcup_{n \in \mathbb{Z}} \{t \in \mathbb{Z} | t_n^A \le t \le t_n^B\},$$

where t_n^A and t_n^B are the nth exit and entrance time of set A and B respectively such that

$$X_{t_n^A} \in A,\ X_{t_n^B} \in B,\ X_t \in (A \cup B)^c \ \ for \ \ t_n^A < t < t_n^B.$$

Definition 2 (Transition Paths [34, 30, 31]). *For a given path $\{X_t\}$, the nth transition path from A to B is*

$$P_n = \{X_k | t_n^A \le k \le t_n^B\}.$$

The set of all transition paths is defined as $\mathscr{P} = \bigcup_{n \in \mathbb{Z}} \{P_n\}$.

The probability distribution of transition paths is defined as:

Definition 3 (Probability of Transition Paths [34]). *The probability of observing transition path at state i is defined as*

$$m_i^{AB} := \lim_{N\to\infty} \frac{1}{2N+1} \sum_{t=-N}^{N} \mathbf{1}_{\{X_t=i\}}\mathbf{1}_{\{t\in\mathscr{T}\}}. \tag{16}$$

and $\mathbf{1}_{\{\cdot\}}$ is the indicator function with $\mathbf{1}_{\{exp\}} = 1$ if the logical variable $exp =$TRUE, and 0 otherwise.

Here m_i^{AB} describes the probability that the cell is on a transition path from A to B and bypassing state S_i.

Definition 4 (First Entrance and Last Exit Time). *Given the Markov chain $\{X_t\}$, the first entrance time τ_A^+ into set A, and the last exit time τ_A^- from set A are defined as*

$$\tau_A^+ := \inf\{t > 0 : X_t \in A\},$$
$$\tau_A^- := \inf\{t > 0 : X_t^R \in A\}.$$

where $X_t^R := X_{-t}$ is the path of time-reversed process of X_t, and X_t^R has the transition probability matrix $\hat{P}_{ij}^R = \frac{\mu_j \hat{P}_{ji}}{\mu_i} = \hat{P}_{ij}$ due to the detailed-balance condition of coarse-grained Markov chain.

Definition 5 (Committor Function [34]). *The forward and backward committor functions are defined as*

$$q_i^+ := \mathbb{P}_i(\tau_B^+ < \tau_A^+), \quad q_i^- := \mathbb{P}_i^R(\tau_B^- > \tau_A^-).$$

Here $\mathbb{P}_i$ denotes the probability of the forward process X conditioned on $X_0 = i$ and $\mathbb{P}_i^R$ the probability of the reversed process X^R conditioned on $X_0^R = i$.

Here q_i^+ denotes the probability that the cell starting from cluster S_i first enters set B rather than set A, and q_i^- the probability that the cell arriving at cluster S_i came last from set A instead of B.

Committor functions can be computed via linear systems.

Proposition 5. *[34] The committor functions solve the following linear equations*

$$\begin{cases} \sum_k \hat{P}_{ik} q_k^+ = q_i^+, & i \notin A \cup B, \\ q_i^+ = 0, & i \in A, \\ q_i^+ = 1, & i \in B. \end{cases}$$

$$\begin{cases} \sum_k \hat{P}_{ik} q_k^- = q_i^-, & i \notin A \cup B, \\ q_i^- = 1, & i \in A, \\ q_i^- = 0, & i \in B. \end{cases}$$

We can check that $q_i^- = 1 - q_i^+$ for the detailed-balance process. Then we can have transition paths probability computed as follows.

Proposition 6. *[34] The probability of transition paths defined in* (16) *can be expressed as*

$$m_i^{AB} = \mu_i q_i^- q_i^+.$$

To observe the transition paths at state i, we pick it with the stationary distribution μ_i, and require that the path last exit from set A and first enter the set B. This happens with the probability q_i^- and q_i^+, respectively.

Similarly, we can define the probability flux of transition paths, which is important to the detection of development trajectories discussed below. It quantifies the proportion of cells that are on a transition path from A to B and moving directly from S_i to S_j.

Definition 6 (Probability Flux of Transition Paths [34])**.**

$$f_{ij}^{AB} := \lim_{N\to\infty} \frac{1}{N} \sum_{t=1}^{N} \Big(\mathbf{1}_{\{X_t=i, X_{t+1}=j\}} \cdot \sum_n \mathbf{1}_{\{t_n^A \le t < t+1 \le t_n^B\}} \Big).$$

We can also write f_{ij} in terms of committor functions which is used in computation

Proposition 7. *[34] The probability flux of transition paths can be expressed as*

$$f_{ij}^{AB} = \mu_i \hat{P}_{ij} q_i^- q_j^+.$$

We therefore define the concept of development trajectories that connect different attractor basins to quantify the cell lineages.

Definition 7 (Development Trajectories). *A development trajectory* $\omega_{dr} = (i_0, i_1, \ldots, i_n)$ *from single-cell sets A to B is a path that passes through cell states connecting A and B without self-interactions such that*

$$i_0 \in A, i_n \in B, i_j \in (A \cup B)^c \text{ and } i_j \neq i_k, \text{ for } 0 \leq j \neq k \leq n.$$

To calculate the effective probability development trajectories, we need to eliminate the effect of detours along transition paths, and therefore define the notion of net transition paths probability flux as follows

Definition 8 (Net Transition Paths Probability Flux). *The net transition paths probability flux of transition paths from state i to j during the transition from set A to B is defined as*

$$f_{ij}^{+} = \max\{f_{ij}^{AB} - f_{ji}^{AB}, 0\}.$$

With the transition paths probability flux, we can define the capacity for each development trajectory (transition path).

Definition 9 (Capacity and Bottleneck [34]). *Given the development trajectory* $\omega_{dr} = (i_0, i_1, \ldots, i_n)$ *from set A to B, its capacity is defined as*

$$c(\omega_{dr}) = \min_{0 \leq k \leq n-1} f_{i_k i_{k+1}}^{+}.$$

For all the possible paths connecting A and B, we can calculate their capacities based on the formula.The relative likelihood of each transition path is defined by the proportion of its capacity to the sum of all path capacities from A to B, as the numbers shown in the main text figures. It can be interpreted as the effective proportion of transition flux along the interested developmental trajectory.

By joining the most probable paths from the predetermined root state S_r^* to all the other states, a tree structure of graph can naturally arise, also known as the shortest path-tree in graph theory. The connecting path between S_r^* and any cell state S_k^* on the tree coincides with the most probable path from S_r^* toward S_k^*. For simplicity and intuition, we will call the computed shortest path-tree as the Most Probable Path Tree (MPPT) that reflects the cell lineage.

In the Python package of MuTrans, we use functions in PyEMMA [35] for the computations in transition path theory.

Identification of Transition Cells.

In addition to the MPPT, which describes the stochastic transitions on the cell states level, we also aim to quantify the cell state transition in the single-cell resolution.

To inspect the transition process from state S_i^* to S_j^*, we first sort the set of cells $\mathcal{R}_{ij} \subset S_i^* \cup S_j^*$ that are relevant to specific process through the threshold h_{mb} of membership matrix, such that $\mathcal{R}_{ij} = \{x \in S_i^* \cup S_j^* | \min\{\rho_i^*(x), \rho_j^*(x)\} > h_{mb}\}$. This step ensures that the cells evolved in the bifurcation to other lineage would be excluded from the analysis of S_i^* to S_j^* transition.

To discover transition cells, we define a cell-specific measure, Transition Cell Score (TCS) $\tau_{ij}(x)$ for $x \in \mathcal{R}_{ij}$,

$$\tau_{ij}(x) = \frac{\rho_i^*(x)}{\rho_i^*(x) + \rho_j^*(x)}.$$

From the OLE dynamical model (1), τ_{ij} remains one or zero when the cell wanders around the attractors of S_i^* or S_j^*, while τ_{ij} transits from one to zero as the cellular state switches from S_i^* to S_j^* along the transition path. Motivated by this intuition, we arrange all the cells in $\mathcal{R}_{ij}$ according to τ_{ij} in descending order, and denote the ordered cells as $x_{ij}^1, x_{ij}^2, \ldots, x_{ij}^L$. Then we define a normalized function $\tilde{\tau}_{ij}(t)$ on $t \in [0, 1]$ such that $\tilde{\tau}_{ij}(t_m) = \tau_{ij}(x_{ij}^m)$ for $m \in \{1, 2, \ldots, L\}$, where $t_m = m/L$. To deal with the noise in data, we also use the logistic function

$$l(t; \theta, t_c) = \frac{1}{1 + e^{\theta(t - t_c)}} \tag{17}$$

to smooth the reordered $\tilde{\tau}_{ij}(t)$, where the parameter θ reflects the steepness of transition.A larger θ corresponds to a sharper transition.

For the smoothed $\tilde{\tau}_{ij}$, we expect to observe a transition layer in its graph (Supplementary Fig. 3), within which the value of $\tilde{\tau}_{ij}$ drops from one to zero. Consistent with the dynamical models description, the cells in transition layer such that $|\tilde{\tau}_{ij} - 0.5| < h_{tl}$ are therefore defined as transition cells $\mathcal{T}_{ij}$ from state S_i^* to S_j^*. The order of transition cells reflects the progress of state transition process. The rest of cells in $\mathcal{R}_{ij}$ are then denoted as stable cells $\mathcal{M}_i$ or $\mathcal{M}_j$, depending on their cluster identity S_i^* or S_j^*.

It is worth noting that the order of transition cells according to TCS is different from the well-known pseudotemporal ordering of the cells, which attempt to align all the cells from data. From stochastic dynamics point of view, the cells that fall deep into the well of attractor basin will fluctuate around the fixed point, and the concept of order is not

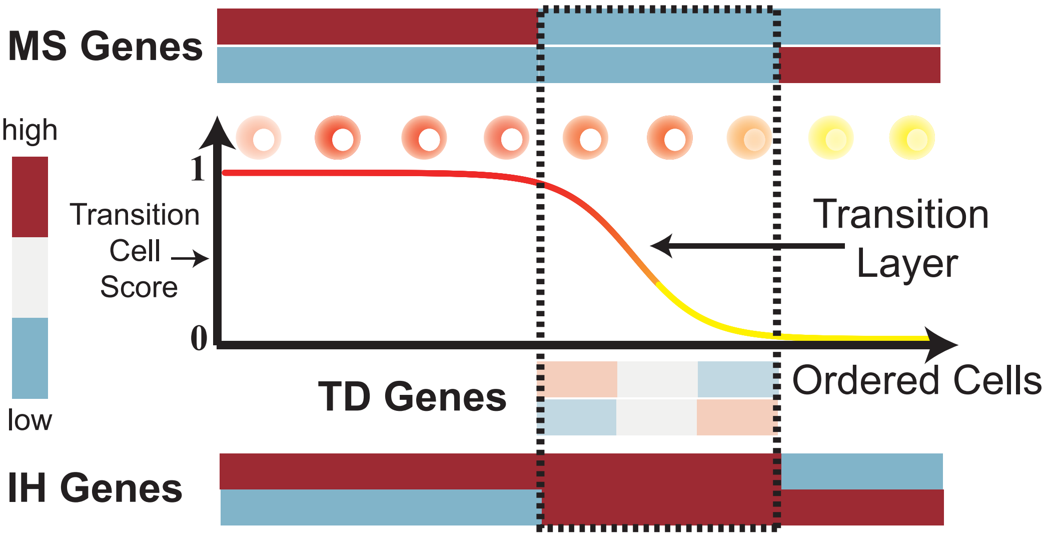

Supplementary Fig. 3: Illustration of transition cell analysis. The cells are ordered by the transition cell score (TCS), where a transition cell layer is expected. The TD, MS and IH genes are then distinguished based on the Transcendental procedure.

well defined for these cells.

Classification of Marker Genes

Based on the calculated TCS and identified transition cells in the switch from S_i^* to S_j^*, the Transcendental further classifies the marker genes regarding transition and stable cells (Supplementary Fig. 3). In the first step, all the differentially expressed (DE) genes between S_i^* and S_j^* are probed via standard approaches. Next, all the DE genes are classified into three types based on the following criterion:

- **Transition-Driver (TD)** Genes, reflecting transient dynamics of transition cells. The expression of TD genes changes dynamically within the transition cells and reflect the progress of cell-fate switch, therefore can be considered as the drivers of transition. To quantify TD genes, we use the correlation between gene expression and TCS in the transition cells set $\mathcal{T}_{ij}$. For each DE gene g, let $g(x)$ denote its expression in cell x. The gene expression and TCS correlation is calculated as

$$\mathcal{C}_g = \frac{\sum\limits_{x\in\mathcal{T}_{ij}} (\tau_{ij}(x) - \bar{\tau}_{ij})(g(x) - \bar{g})}{\sqrt{\sum\limits_{x\in\mathcal{T}_{ij}} (\tau_{ij}(x) - \bar{\tau}_{ij})^2}\sqrt{\sum\limits_{x\in\mathcal{T}_{ij}} (g(x) - \bar{g})^2}}$$

  where the averages $\bar{\tau}_{ij}$ and $\bar{g}$ are taken over all the transition cells. A larger value of $|\mathcal{C}_g|$ suggests that the expression of g is more accordant (either positively or negatively) with the cell-fate transition dynamics by TCS. Hence we pick all the

DE genes with $|\mathcal{C}_g| > h_{TD}$ where h_{TD} is an adjustable threshold, and define the selected genes as TD genes during transition. The TD genes are ranked based on the absolute values of correlation coefficients calculated.

- **Meta-Stable (MS)** Genes, distinguishing the stable cells uniquely. Aside from dynamical TD genes that highlight the transition cells, Transcendental next probes the DE genes that are distinctively expressed in stable cells. To this end, for each DE gene that is non-TD, we perform two groups of DE analysis on $\mathcal{M}_i$ v.s. $\mathcal{T}_{ij}$ and $\mathcal{M}_j$ v.s. $\mathcal{T}_{ij}$ respectively. The significantly up-regulated genes in $\mathcal{M}_i$ or $\mathcal{M}_j$ are termed as MS genes, exclusively mark the stable cells in S_i^* or S_j^*. MS are ranked based on their significance in DE analysis and the top MS genes tend to express uniquely in stable cells than transition cells.

- **Intermediate-Hybrid (IH)** Genes, demonstrating the mixed cell-fate identity of transition cells. The rest DE genes that are non-TD and non-MS, are termed as IH genes. The IH genes express eminently both in the transition cells and in the stable cells from one specific cluster (S_i^* or S_j^*), suggesting the resemblance of transition cells with both cell-fate during the transition. In the sense of IH genes expression, the transition cells are the mixture of S_i^* and S_j^* states cells. The IH genes are ranked based on their significance in the DE analysis to detect MS genes, but with the reversed order. The top IH genes tend to express uniformly in both stable and transition cells.

Overall, above classification of genes resolves the previous discrepancy in defining transition cells, either from the dynamical [6] or the static [7] perspectives. We argue that the transition cells may display both transient (dynamical) and hybrid (static) features, manifesting in the expression of TD or IH genes respectively.

Directionality of Transition

In Transcendental, the directionality of transition is indicated by the tree obtained in lineage inference. We note that reverting the starting and targeting state does not change the identification results of transition cells or genes, since we have the relationship for TCS that $\tau_{ij}(x) = 1 - \tau_{ji}(x)$ and the metric of transition cells or genes defined above are invariant under such transformation.

DECLARE Pre-Processing Module for Large-Scale Dataset

The rapid emerging of large-scale scRNA-seq datasets poses computational challenges to the trajectory analysis. Therefore we propose an optional pre-processing module here, named DECLARE (dynamics-preserving cells aggregation) to first coarse-grain the transition dynamics on the scale of microscopic attractors instead of single-cells to reduce the computational complexity.

The construction of rwTPM among microscopic attractors is inspired by the concept of coarse-graining of Markov Chain defined below.

Definition 10 (Coarse-graining of Markov Chain)**.** *Given cell-cell scale Markov chain* $\{x_t\}$ *on the state space* S *with transition probability matrix* $\mathbf{P} = \{p(x, y)\}$ *and a partition* $M = \cup_{k=1}^{K_m} M_k$ *of microscopic attractors, the coarse-graining of* $\{x_t\}$ *is defined as a Markov chain* $\{X_t\}$ *on the state space* $\{M_k\}_{k=1}^{K_m}$ *with transition probability matrix*

$$T_{ij} = \lim_{N\to\infty} \frac{\sum_{t=1}^{N} \mathbf{1}_{\{x_t \in M_i, x_{t+1} \in M_j\}}}{\sum_{t=1}^{N} \mathbf{1}_{\{x_t \in M_i\}}}, \tag{18}$$

where the limit is taken in the almost sure sense, and $\mathbf{1}_{\{.\}}$ *is the indicator function.*

In DECLARE, we begin with the partition of the state space into $M_k$with the clustering of cells in hundreds or thousands of clusters, using methods such as k-means or kNN network partition. Next, the coarse-grained transition probability matrix can be indeed calculated analytically instead of through numerical simulations, which is guaranteed by the following proposition:

Proposition 8. *[34] The coarse-grained random walk transition probability matrix (cg-rwTPM) defined by* (18) *can be expressed by cell-cell transition probabilities* $p(x, y)$ *and its stationary distribution* $\pi(x)$*,*

$$T_{ij} = \frac{\sum_{x\in M_i} \sum_{y\in M_j} \pi(x)p(x, y)}{\sum_{x\in M_i} \pi(x)}.$$

It is also straightforward to verify that the coarse-grained Markov chain X_t with $\{T_{ij}\}$ has the stationary distribution $\boldsymbol{\mu}$ with $\mu_i = \sum_{x\in M_i} \pi(x)$.

With the calculated T_{ij} from DECLARE, we can then take it as the input to the MuTrans as the cell-cell scale rwTPM, to construct dynamical manifold and transition

trajectories.

Adjustable Parameters in MuTrans Implementation

Despite that the MuTrans results are consistent with dynamical system model, it does *not* directly fit the model parameters or simulate the model trajectory as what has been done in [36]. This feature reduces the degree of freedom in algorithm and saves computational costs of MuTrans. As shown in Supplementary Table 1, the majority of parameters in MuTrans, mainly involves 1) constructing original cell-cell random walk based on the Gaussian kernel 2) specifying the number of clusters (attractors) or number of micro states in DECLARE and 3) the threshold to determine transition cells or genes.

**Parameters**	**Meaning**	**Default**
$m(x, y)$	metric in Gaussian kernel	Euclidean
perp	perplexity parameter in determining Gaussian kernel	$N_{cell}/2.5$
h_θ	threshold of average sharpness parameter to construct the neighbor graph for MPPT lineage inference	
h_{2D}	threshold of membership function in fitting local mean and variance of dynamical manifold	0.5
h_{mb}	threshold of membership function to include in the interested transition process	0.5
h_{tl}	threshold of difference between TCS and 0.5 to define transition cells	0.45
h_{TD}	threshold of correlation to determine TD genes	0.5

Supplementary Table 1: The major adjustable parameters in MuTrans and Transcendental procedure.

The Choice of Attractor Numbers

The determination of cluster numbers remain the challenging problem in single-cell transcriptome data analysis. In MuTrans implementation, we suggest the following strategies to choose the number of attractors:

- Given the constructed cell-cell rwTPM, we can calculate the eigenvalues and use the eigen-peak index (EPI) to provide the reference of attractors number. Suppose that we have the eigen-values of symmetric cell-cell weight matrix $w(x, y)$ and

degree $d(x)$, we then sort the eigen-values of normalized weight matrix $D^{-\frac{1}{2}}WD^{-\frac{1}{2}}$ as $|\lambda_1| > |\lambda_2| > \cdots > |\lambda_N|$. The $k-$th EPI is defined as

$$EPI_k = \frac{\lambda_k^2}{\lambda_{k+1}^2},$$

When a peak of the EPI is observed at index k, we reason that there is an obvious eigen-gap in the normalized graph Laplacian of the cellular network, and therefore k is a candidate for the number of clusters. Note that we may observe multiple peaks, which means that we may choose different k depends on desired our resolutions.

- For assessment, after the implementation of MuTrans, we can inspect the re-ordered rwTPM across cell-cell, cluster-cluster, and cell-cluster scales, with cells within the same attractor are aligned together. Ideally, the re-ordered rwTPMs should present clear block structures since the cellular transition probabilities within the same attractor are significantly greater than those among cells from different attractors.
- We also combine with prior biological knowledge, marker genes analysis, or compare with labels in original publication to further validate the results.

Timing and Computation Efficiency of Algorithm

In implementation, the major computation cost of MuTrans involves solving the optimization problem Eq.(9) with number of unknowns (i.e. $\rho_k(x)$) $N * k$, where N is the total number of cells and k is the number of cell clusters. As shown in Supplementary Table 2 , the upper bound for time complexity of MuTrans is $O(N^2k^2)$, which is of the same order in terms of cell number N with other widely applied clustering or dimension reduction algorithms (e.g. original tSNE without acceleration). The major obstacle to boost MuTrans is that the computation complexity also relies quadratically on the number of cluster k.

**Dataset**	**Number of Input Cells**	**Number of Clusters**	**Run Time(s)**
Simulation	2000	2	31
iPSC	1081	9	269
Lymphoid	2018	10	1328
Human HSC	4118	5	792

Supplementary Table 2: Running time of MuTrans for different dataset on personal computers. The tolerance for the first order condition in optimization problem is set as 10^{-6} for all datasets.

In practice, we can adopt the following strategies to speed up the algorithm:

- Coarse-graining the data. For very large dataset, one can apply the DECLARE module to coarse-grain the data. This will decrease N and k simultaneously in the computation. Our benchmark in human HSC dataset (main text Figure 6) shows that the strategy may speed up the computation by one magnitude, while retain accurate dynamical manifold construction and trajectory analysis.

- Adjusting the stopping criterion in the optimization problem. The default setting of MuTrans is that the norm of gradient in objective function should be less than tolerance value 10^{-6}. In practice, we find increase the tolerance can boost the convergence of optimization greatly, without the large change in results regarding transition cells and genes. For example, as shown in Supplementary Table 3, when the tolerance is set as 10^{-5}, the computation time is only one third of the original time. Out of 25 top identified TD, IH and MS genes in Pre-M to M transition, only 2 genes are not consistent with the results under 10^{-6} tolerance setting. When setting the tolerance as 10^{-4} , the computation time efficiency is boosted by 9 fold, with still more than 75% top TD,IH and genes are consistent with the previous findings. This observation also suggests the robustness of MuTrans to probe transition cells and genes.

**First Order Tolerance**	**Run Time(s)**	**Number of Inconsistent Top Genes**
10^{-6}	269	
10^{-5}	91	2
10^{-4}	38	6

Supplementary Table 3: Effect of changing first order tolerance on algorithm performance in iPSC dataset.

Supplementary Note 3: Data Analysis Details

The Simulation Dataset

To test consistency of our method with models of multi-scale dynamics, we simulate the stochastic state-transition process based on 1)a saddle-node bifurcation model in the regime of intermediate noise level 2) the over-damped Langevin systems confined in multiple potential wells. The full scripts to simulate the data is available at `https://github.com/cliffzhou92/MuTrans-release` with two Matlab live script notebooks provided for reproducible analysis results.

Saddle-node Bifurcation

The model assumed that characteristic gene expression level of the cell x_t was confined by the conceived potential field $V(x,\lambda)$ from the gene regulatory network, which was influenced by both the extrinsic signal λ and stochastic noise with amplitude σ.

$$\begin{cases} dx_t = -\partial_x V(x_t,\lambda)dt + \sqrt{2\sigma}dW_t, \\ \frac{d\lambda}{dt} = 1. \end{cases}$$

Here we chose the potential function $V(x,\lambda) = 100\left(\frac{1}{4}x^4 - \frac{3}{2}x^2 + \lambda x\right)$ and the noise level $\sigma = 40$. As shown in Figure 2a of main text,the saddle-node bifurcation occurs at $\lambda = -2$ and $\lambda = 2$, and within the range $\lambda \in [-2, 2]$ the systems exhibit two stable fixed points and one unstable saddle-point, which correspond to the stable and transition cell state respectively.

As shown in the blue-colored simulation trajectory in Figure 2a of main text, the presence of noise in the gene expression drives the cell state switch prior to the bifurcation point at $\lambda = 2$.

To generate simulation data, we used Euler-Maruyama method to solve the SDEs with initial condition $x_0 = 2.1038$, time step 10^{-5}, and took the 2,000 cells near phase-transition points on the bifurcation plot to construct rwTPM as the input to MuTrans. The script to reproduce simulation and analysis for this dataset is available at `https://github.com/cliffzhou92/MuTrans-release/blob/main/Example/example_saddle_node.mlx`

Back-and-forth Transitions in Potential Fields

To have back-and-forth transitions among potential wells, we simulated the over-damped Langevin systems with sufficiently large noise σ

$$d\mathbf{X}_t = -\nabla V(\mathbf{X}_t)dt + \sqrt{2\sigma}d\mathbf{W}_t,$$

Here the potential filed $V(x)$ has multiple local minimums representing different attractors of dynamical system. Specifically we chose

- Double-Well Potential. $V(x_1, x_2) = \frac{5}{2}(x_1^2 - 1)^2 + 5x_2^2$, where we have two stable points $(-1, 0)$ and $(1, 0)$, and one saddle point in-between $(0, 0)$.

  We chose noise amplitude $\sigma = 2$ and simulated system with Euler-Maruyama methods of time step 10^{-2} and initial condition $x_0 = (1, 0)$. 2,001 samples from simulation trajectories are given as the snapshot input to MuTrans.

  From Supplementary Fig. 4(c), we observe the obvious peak at $K = 2$, and the attractor membership function has the gradual transition through saddle points as shown in (d). The script to reproduce simulation and analysis for this dataset is available at `https://github.com/cliffzhou92/MuTrans-release/blob/main/Example/example_double_well.mlx`

- Tipple-Well Potential.

$$V(x_1, x_2) = 3\exp(-x_1^2 - (x_2^2 - \frac{1}{3})^2) - 3\exp(-x_1^2 - (x_2^2 - \frac{5}{3})^2)$$
$$- 5\exp(-(x_1^2 - 1)^2 - x_2^2) - 5\exp(-(x_1^2 + 1)^2 - x_2^2) + 0.2x_1^4 + 0.2(x_2 - \frac{1}{3})^4$$

  The three stable points are around $(-1.05, 0.04)$, $(1.05, 0.04)$ and $(0, 1.54)$, the three order-1 saddles between attractor basins are $(-0.6, 1.1)$, $(0.6, 1.1)$ and $(0, -0.3)$, and the order-2 saddle (maxima) is at $(0, 0.52)$.

  We simulated system with Euler-Maruyama methods of time step 10^{-2} and initial condition $x_0 = (-1, 0)$. 2,001 samples from simulation trajectories are given as the snapshot input to MuTrans. From Supplementary Fig. 4(f), we observe the obvious peak at $K = 3$.

We let noise amplitude σ change to generate different datasets, and re-run MuTrans to inspect the coarse-grained transition probabilities. The main text results corresponded to $\sigma = 0.8$. With the increase of noise level, the transition probabilities between different attractors (off-diagonal elements) become larger. In addition, the direct transition from attractor 1 to 3 becomes more frequent than the transitions involving attractor 2. These observations are consistent with previous studies about this triple potential-well system [37]. The script to reproduce simulation and analysis for this dataset is available at `https://github.com/cliffzhou92/MuTrans-release/blob/main/Example/example_triple_well.mlx`

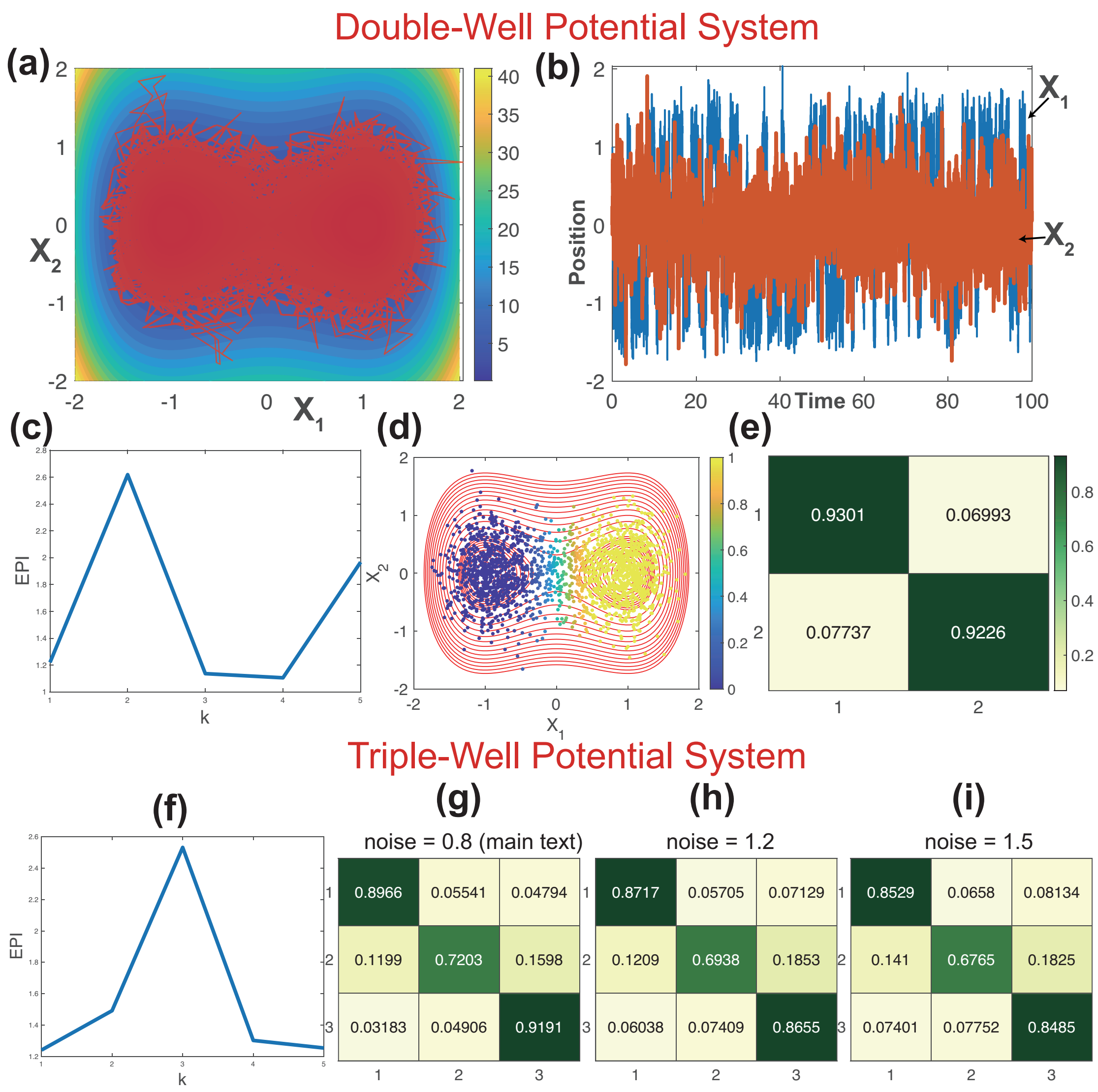

Supplementary Fig. 4: Supplementary for main text Figure 2.(a-b) Simulation trajectories in double-well potential field and time-series display. (c) EPI index. (d) Membership function of right attractor calculated by MuTrans. The cells near saddle point have around 0.5 values. (e) Coarse-grained rwTPM. (f) EPI index for triple-well dataset shown in main text Figure 2. (g-i) Coarse-grained rwTPM with noise amplitude $\sigma = 0.8, 1.2, 1.5$.

The EMT Dataset

The EMT data analyzed in main text was from GSE110357 with 354 cells sequenced by Smart-Seq2 platform. Prior to MuTrans analysis, we selected 1500 highly variable genes using vst method in Seurat pipeline. Following the strategy in original publication [38], cells with large portion of ERCC gene expression (more than 2 percent of all gene counts) are filtered out .We further kept cells with least 200 genes expressed and 100,000 counts. The log-transformed and scaled data is then taken as the input to MuTrans. The Jupyter notebook file to reproduce analysis results for EMT data is available at `https://github.com/cliffzhou92/MuTrans-release/blob/main/Example/example-emt-raw.ipynb`. For this dataset, UMAP was computed based on 50 neighbors in Scanpy.

Guided by EPI (Supplementary Fig. 5(b)), MuTrans found five attractors in the dataset as depicted in (c). Compared with Leiden clustering results (a), MuTrans detected two attractors in mesenchymal cells. Marker gene analysis (d-e) found that each attractor expressed unique genes. Inspecting the known E/M/hybrid states markers (f) in original publication [38], we found that attractor 3 expressed pure epithelial marker Epcam (therefore annotated as E), attractor 0 and 4 expressed pure mesenchymal makers MMp19 and Aspn, attractor 1 express both E hybrid marker Krt5 and M hybrid markers Zeb2/Prrx1 (denoted as Intermediate Cell State 1, ICS1), and attractor 2 express M hybrid markers (denoted as ICS2).

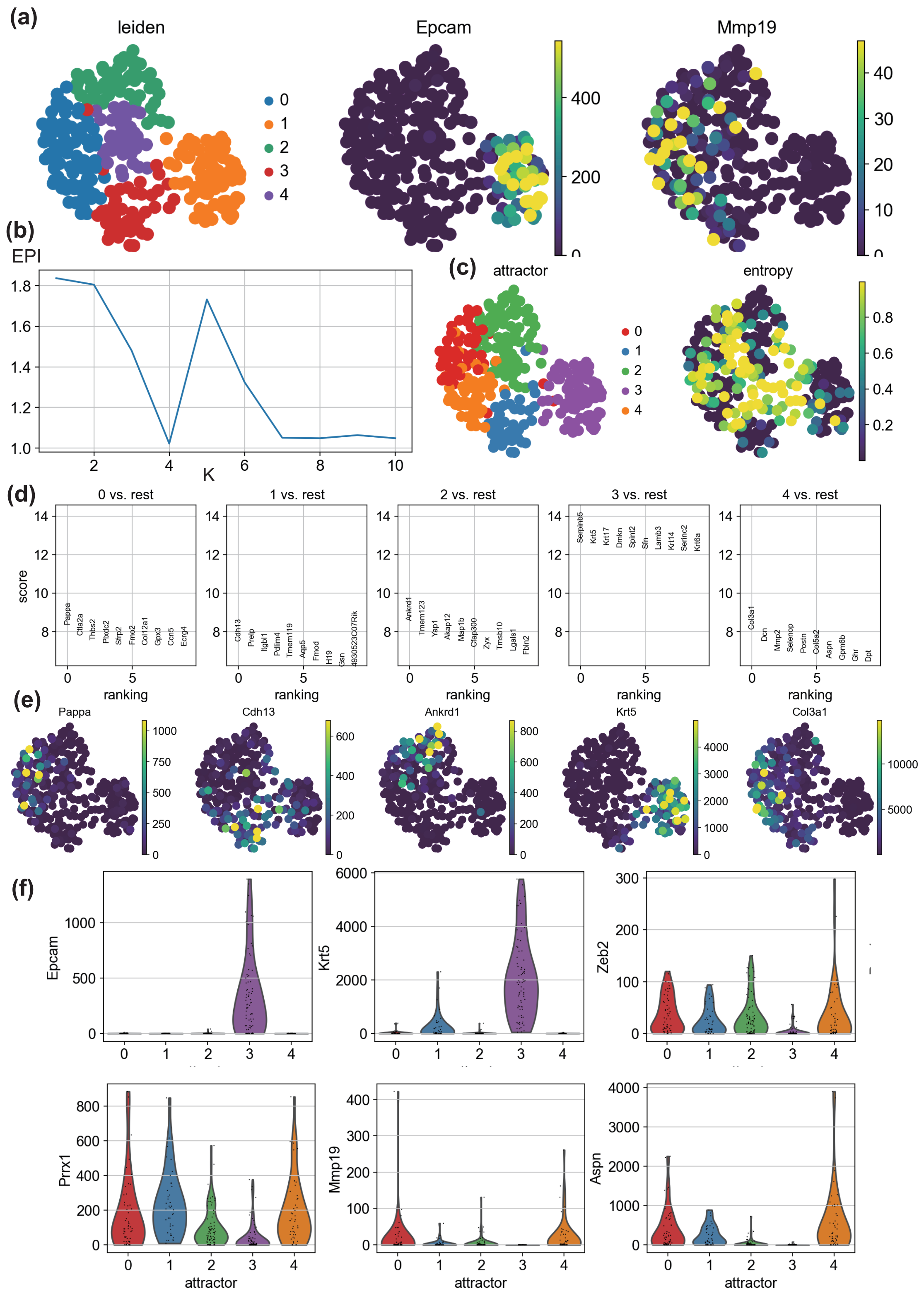

Supplementary Fig. 5: Supplementary analysis results of EMT dataset to main text Figure 3. (a) Leiden clustering results of EMT dataset with UMAP projection. (b)The EPI (eigen-peak index) for this data. We observe an apparent peak at $k = 5$. (c) Attractors and transition entropy calculated by MuTrans. (d-e) Marker gene analysis and plot of each MuTrans attractor.(f) Violin plot of previously known markers for E/M/hybrid states in each attractor, annotating the cell type.

The Early iPSC Differentiation Dataset

The iPSC data analyzed in main text was from `https://www.pnas.org/highwire/filestream/29285/field_highwire_adjunct_files/1/pnas.1621412114.sd02.xlsx`. We analyzed the 1,081 cells collected in first three days of induction experiment to inspect bifurcation dynamics with data log transformed. The processed input gene expression matrix and script to reproduce all the supplementary results for iPSC data is available at `https://github.com/cliffzhou92/MuTrans-release`. The UMAP dimensional reduction of this data was computed in Scanpy with 60 neighbors.

In Supplementary Fig. 6, we displayed the MuTrans results with more details. In UMAP projection of the data, we showed cells colored by MuTrans probed attractors ($K = 9$, corresponds to the apparent peak in EPI (g)) versus the Leiden clustering results with default resolution parameter, where seven clusters were found. The difference was mainly about the additional attractors in epiblast and ectoderm defined by MuTrans in the earliest stage, while other cell types were quite consistent with Leiden, reflecting the difference of resolutions.We also presented the cell lineage inferred by Most Probable Path Tree (MPPT) or transition paths analysis.

To show the robustness toward choosing the number of attractors K, we re-ran MuTrans with $K = 7$ (which is another peak shown in EPI (g)), as results displayed in Supplementary Fig. 6 (h). Interestingly, the attractors are highly consistent with Leiden clustering results in (c). The results regarding transition paths and transition entropy trend during the bifurcation is similar to $K = 9$ as shown in (e-f) and main text Figure 4f.

Next, in Supplementary Table 4 we listed the MS, IH and TD genes during Pre-M to M commitment, used in plotting the trend in Figure 4h of main text. The top enriched GO terms for each group of genes were obtained from `http://geneontology.org/`. In Supplementary Fig. 7, we plotted the most significant M state MS genes TBX2 and ISL1 (which only highly-expressed in stable cells of M state), as well as the top-rank IH gene TBX20 (expressed both in transition cells and stable cells of M state). In (d), we used violin plot to show the difference of gene expression distribution between MS and IH genes.

Finally, we summarized the overall gene expression patterns in the En/M bifurcation from PS state as in Supplementary Fig. 8. Within the PS attractor basin, we further classified three types of cells based on their membership functions: 1) the stable PS cells 2) the cells that have larger membership in Pre-M attractor (called Pre-M-bifur

cells) 3) the cells that have larger membership in Pre-En attractor (called Pre-En-bifur cells). With the genes expression statistical test for each group of cells (the test also including "negative markers" that are uniquely down-regulated), we found certain genes that might be used to predict the cells fate into either Pre-En or Pre-M attractor, and denoted them as bifur-prediction genes in PS attractor.

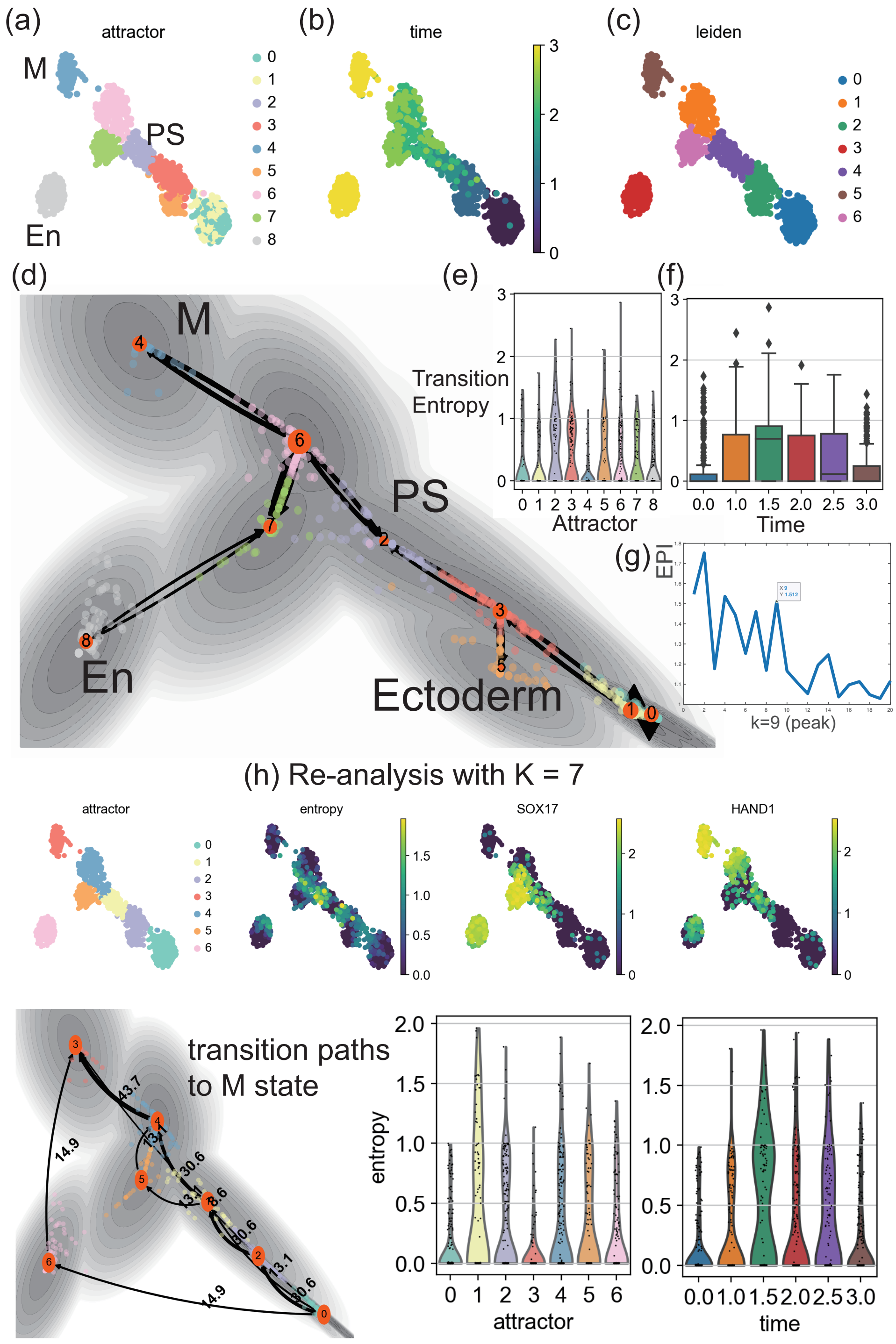

Supplementary Fig. 6: Dynamical system analysis of early iPSC differentiation data by MuTrans. (a-c) UMAP projection of the dataset with cells colored by MuTrans attractors ($k = 9$), collection time and Leiden clustering results. (d) Inferred maximum probability flow tree (MPFT) as cell lineage. (e) Violin plots of MuTrans cell transition entropies in each attractor. (f) Box plots of MuTrans cell transition entropies (n = 1081 cells) in each cell collection time, corresponding to the violin plot in main text Figure 4f. Box plots indicate median (middle line), 25th, 75th percentile (box), 5th and 95th percentile (whiskers) and the outliers (single points). The number of cells in each box (collection times) are n =231, 166, 93, 211,122 and 258 respectively. (g) EPI index of the dataset. (h) Re-analysis of the dataset with k selected as 7.

Genes Group	Genes Name	Top GO Terms
Pre-M-state MS genes	T NANOG MIXL1 SOX17	endodermal cell differentiation; endoderm formation
Pre-M-state IH genes	KIT VEGFA LEFTY1 NOTCH1 NOTCH2 PDGFB PDGFA	positive regulation of MAPK cascade; positive regulation of phosphatidylinositol 3-kinase signaling
TD genes (down-regulated)	PARD3 FOXC1 NUMB FGFR1 FOXH1 TUBB TGFB1 GATA4 SIRPA FGF8 FGF10 BMPR1A TGFBR2 BMP2 FZD7 PTX1 FZD1 ACVR1B DKK1 FZD4 ENG BMPR2 EVX1 ALCAM ACVR2A EOMES EPCAM GSC GATA6 RPL35A PTX2	negative regulation of cardiac muscle cell differentiation; negative regulation of cell fate commitment
M-state IH genes	TBX20 KDR WNT4 EMILIN2 TGFB2 PTCH1 INHBA HAND2 BMP4 MYL4 SFRP1 PDGFRB FZD2 HAND1 MSX1	anatomical structure formation involved in morphogenesis; heart morphogenesis; muscle structure development
M-state MS genes	MSX2 MYL3 GATA5 WNT11 ISL1 TBX2	cardiac muscle cell myoblast differentiation; regulation of transcription involved in heart development; cardioblast differentiation; endocardial cushion morphogenesis; outflow tract septum morphogenesis

Supplementary Table 4: (Supplementary to the gene expression trend in Figure 4h of main text) The list of MS, IH and TD genes in Pre-M to M transition of iPSC data. The genes marked with red color are top-rank, significant genes used to calculate gene expression trend in Figure 4h of main text. The selected GO terms for different groups of genes are also displayed.

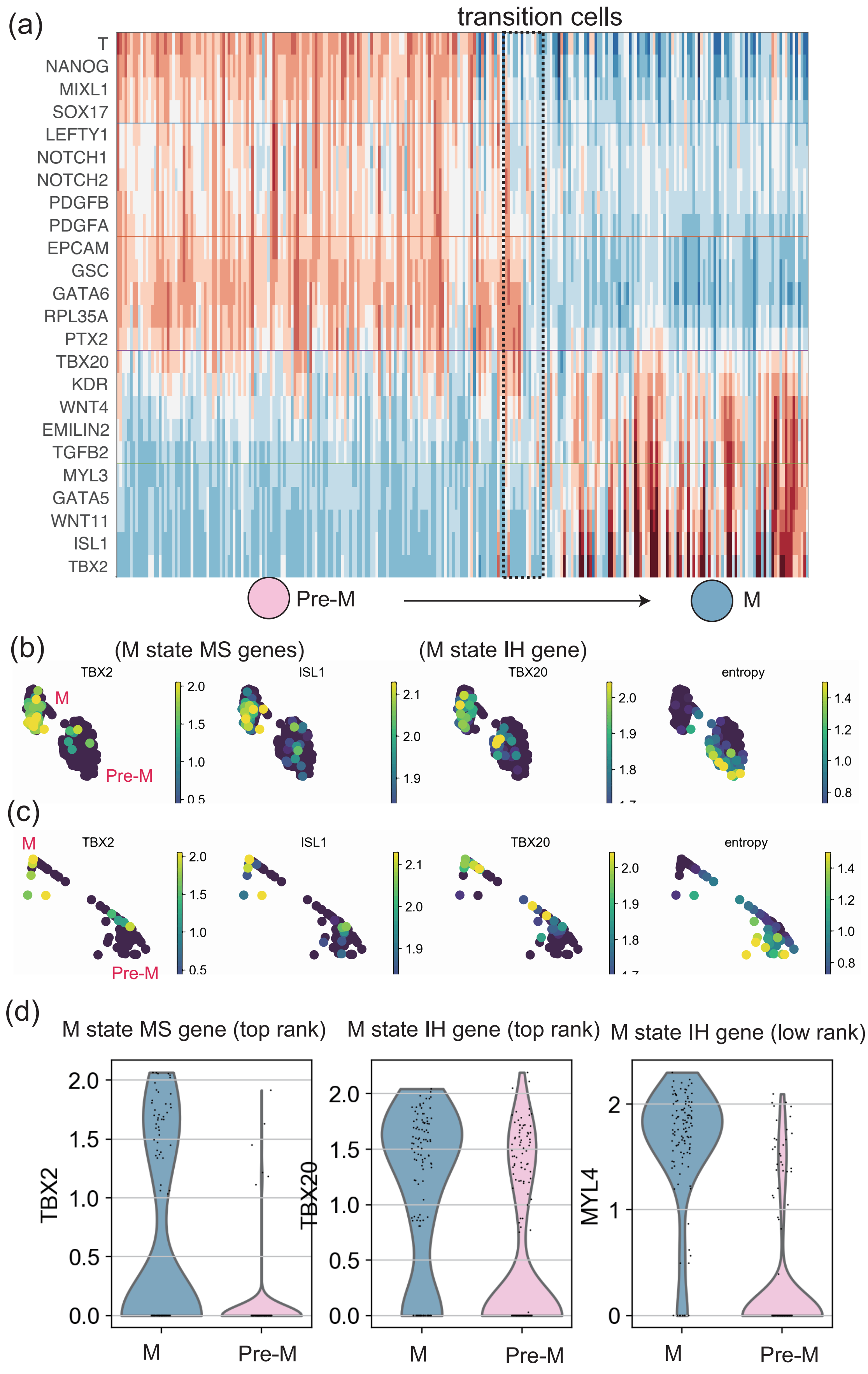

Supplementary Fig. 7: (a) The heatmap of top 5 genes expression for each group as selected in Supplementary Table 4. (b-c) Expression of selected genes plotted in UMAP and MuTrans transition coordinates projection. (d) Gene expression violin plots of selected genes.

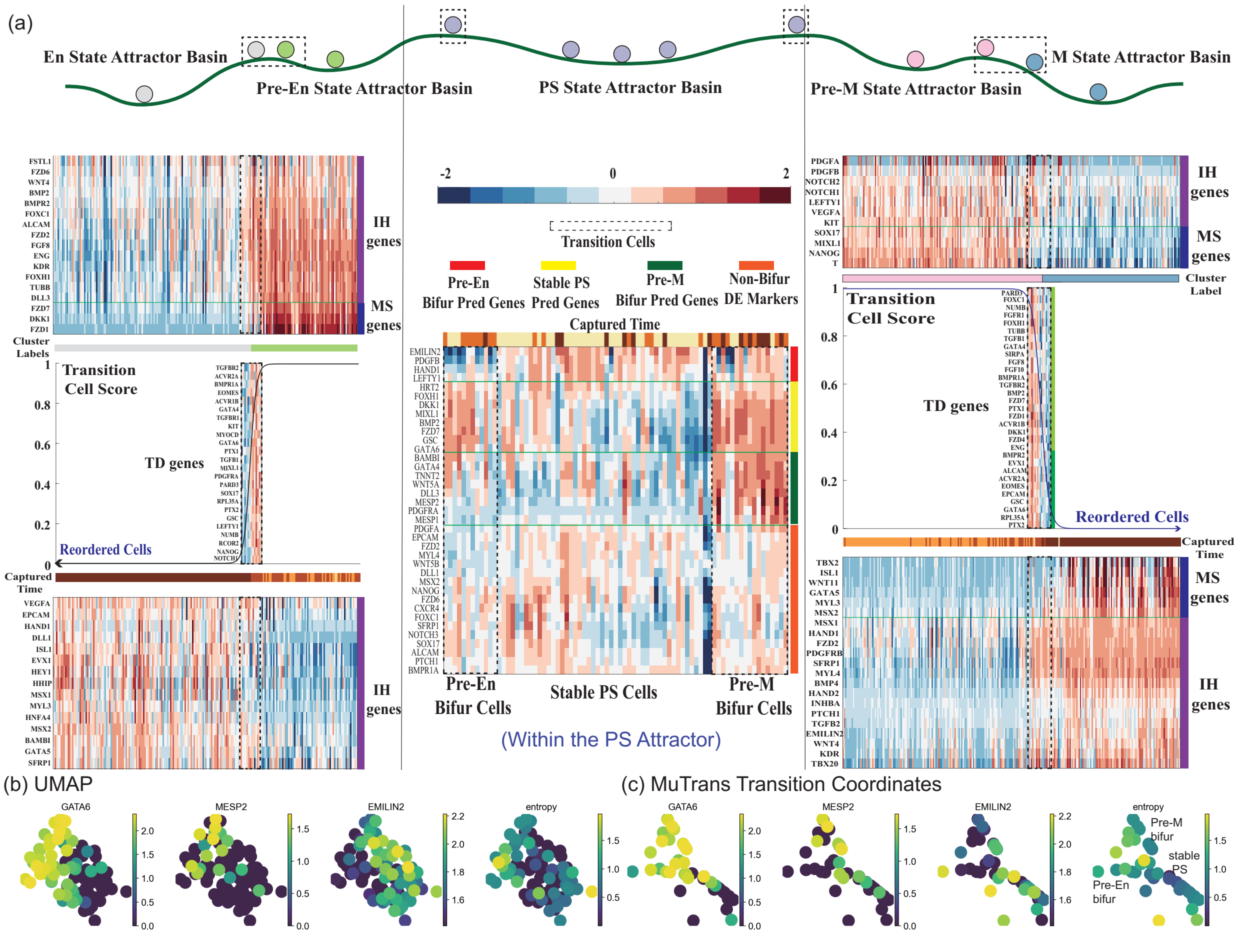

Supplementary Fig. 8: Overall gene analysis toward the bifurcations into En/M states of iPSC data. (a-top) The schematic illustration of the dynamical manifold during bifurcations. (a-bottom left) The expression heat map of MS, IH and TD genes during Pre-En to En transition. The positions of cells (horizontal axis) are aligned with the schematic dynamical manifold. (a-bottom middle) The gene expression heat map within the bifurcating PS state. The cells are arranged by transition cells toward En lineage (left), stable PS cells(middle) and transition cells toward M lineage(right). The genes are sorted to predict the different groups of cells. (a-bottom right) The expression heatmap of MS, IH and TD genes during Pre-M to M transition. (b-c)Expression of selected PS bifur-pred genes plotted in UMAP and MuTrans transition coordinates projection. Within PS attractor, down-regulation of GATA6 distinguishes the stable cells with bifurcating cells. Up-regulation of MESP2 predicts the toward-Pre-M bifurcating cells, and down-regualation of EMILIN2 predicts the toward-Pre-En bifurcating cells.

The Myelopoiesis Dataset

The Myelopoiesis data analyzed in main text was from GSE70245, containing 382 cells sequenced by Fluidigm C1 platform. For comparable analysis with labels in original publication, we downloaded the 532 feature genes identified by ICGS from https://static-content.springer.com/esm/art%3A10.1038%2Fnature19348/MediaObjects/41586_2016_BFnature19348_MOESM464_ESM.xlsx as the input to MuTrans. We used tSNE dimensional reduction to remove seven outlier cells. The data matrix is then log transformed. The script for data processing can be downloaded at https://github.com/cliffzhou92/MuTrans-release/blob/main/Data/myelopoiesis_process.m.

The processed input gene expression matrix and script to reproduce all the supplementary results for EMT data is also available at https://github.com/cliffzhou92/MuTrans-release.

We displayed the dynamical system analysis results in Supplementary Fig. 9 and dynamical manifold in Supplementary Fig. 10. For myelopoiesis dataset we used the tSNE dimension reduction results as the input 2D embedding to construct transition coordinates and the subsequent landscape. In Supplementary Fig. 10 (c-e), we compared the discrepancy between MuTrans results and the original labels generated by ICGS method in [39], and find the difference mainly stemmed from the transition cells recognized by MuTrans. The ICGS clustering tends to assign transition cells into either of the attractors during cell-fate switch.

In Supplementary Table 5 and Supplementary Fig. 11, we provided the Transcendental genes list during Multi-Lin to Gran transition as the supplementary to Figure 5c of main text. The Transcendental results for HSPC to Meg transition was also displayed in Supplementary Fig. 12 and Supplementary Table 6.

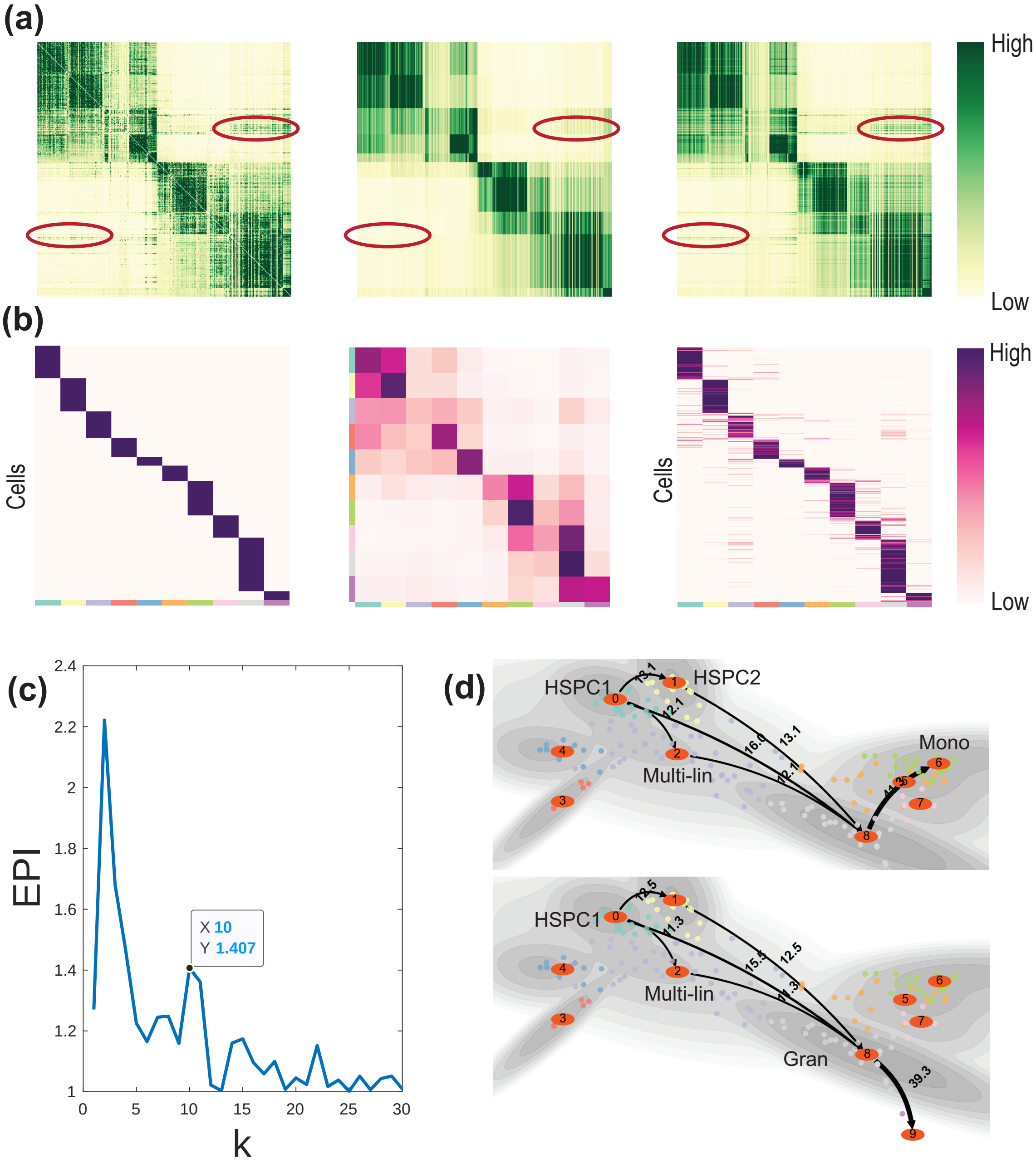

Supplementary Fig. 9: Dynamical system analysis of myelopoiesis data by MuTrans. (a) The rwTPMs constructed from cell-cell (left), cluster-cluster (middle) and cell-cluster (right) resolutions. Note that while cluster-cluster resolution keeps the main block structure of original cell-cell resolution rwTPM, the cell-cluster resolution TPM recovers finer details (illustrated by the red circles). (b) The obtained cell-cluster assignment (left), cluster-cluster transition probability matrix (middle) and cell-cluster membership matrix (right) through optimization, which jointly induce the optimal rwTPMs at cluster-cluster and cell-cluster resolution in (a). (c) The EPI (eigen-peak index) for this data. We observe an apparent peak at $k = 10$, which is the cluster number we choose in MuTrans for this dataset. (d) The inferred transition paths from HSPC to gran or mono cell fates.

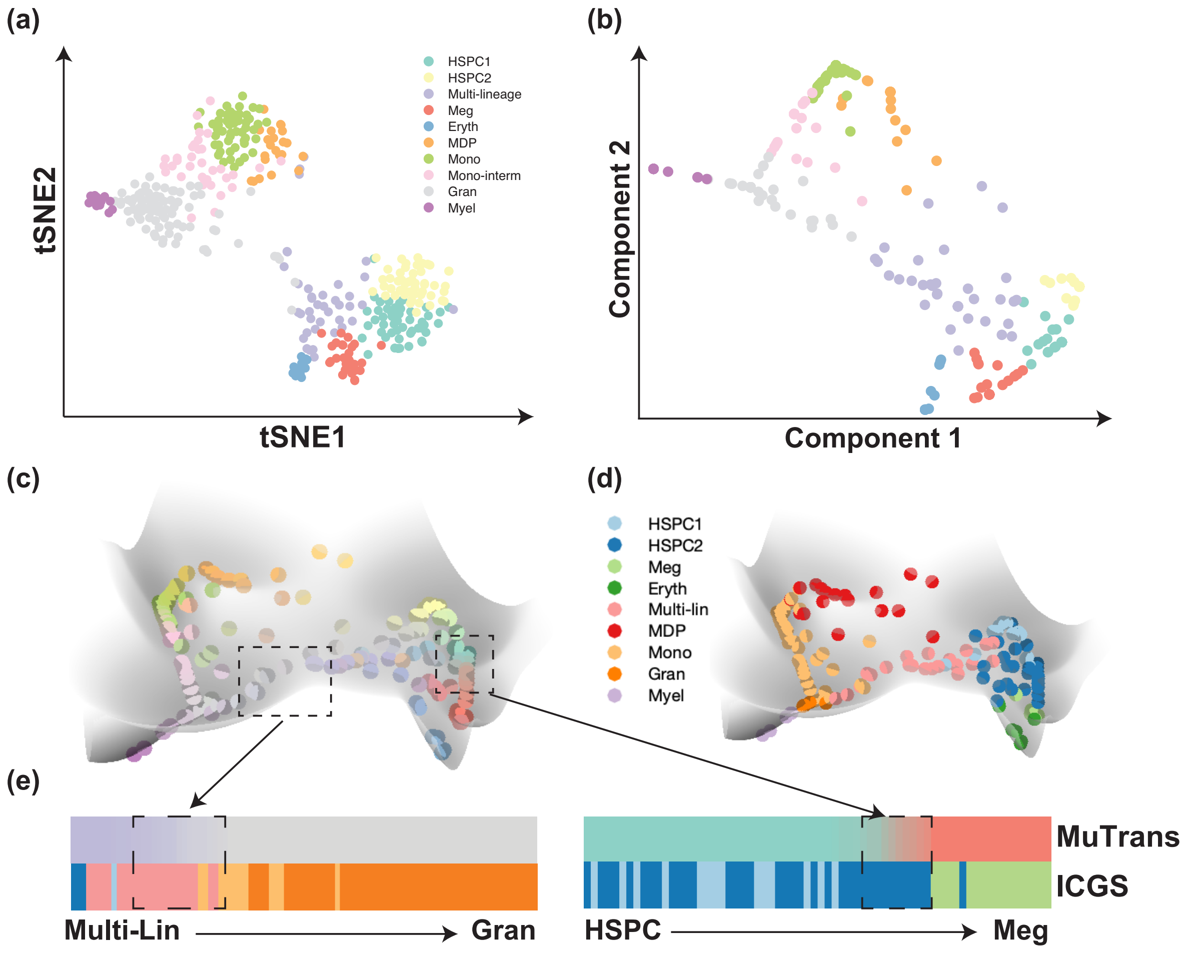

Supplementary Fig. 10: Dynamical manifold analysis of myelopoiesis data. (a) The two-dimensional embedding of single-cell data constructed from the eigen-space of weighted rwTPMs, colored by MuTrans coarse-grained clusters. (b) The constructed transition coordinates ξ as the input to dynamical manifold, based on averaging the two-dimensional embedding in (a) by membership matrix . (c) The constructed dynamical manifold with cells colored by their membership in different clusters, and transition cells in Multilin-Gran and HSCP-Meg transition marked by dashed squares. (d) The dynamical manifold with cells colored by the ICGS labels in original paper. (e) Comparison of MuTrans cell label (colors displayed by cell membership in the clusters) and ICGS label in original publication. The transition cells are marked with dashed squares. In ICGS clustering, the transition cells are assigned into either of the attractors in cell-fate switch.

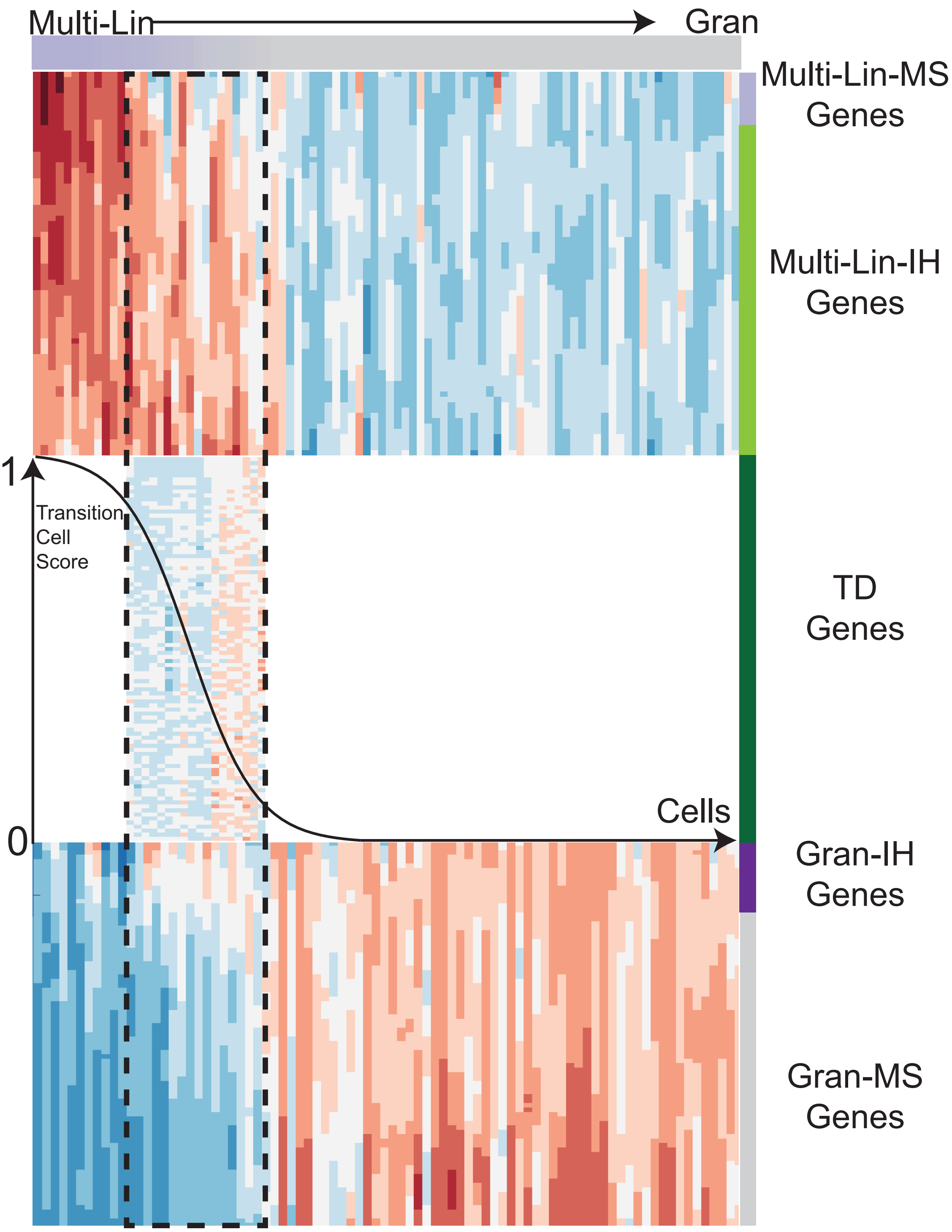

Supplementary Fig. 11: (Supplementary to Figure 5c of main text)Transcendental results of myelopoiesis data for Multi-lin to Gran cell transitions.

Genes Group	Genes Name
MultiLin-state MS genes	Car2 Ctla2b Muc13 Slc22a3 Pitpnc1 Zfp488 Vamp5 Vezf1 Mfsd2b Apoe
MultiLin-state IH genes	Slc45a3 Tgtp2 Angpt1 Zfp36l2 Egr1 Ctla2a Ptrf Rgs1 Smim5 Zfp9 Jun Zfp65 Myct1 Rbp1 Cpa3 Olfr613 Gnb1 Malat1 H2-Q4 Fos Gata2 A130077B15Rik Dpysl2 Dusp1 Fyb Gcnt2 Zfp36 Calcrl Meis1 Gpr56 Spns2 Gm17821 Samsn1 Gm16702 Tmsb10 Tspan13 Syt11 Ifitm3 Tmem176b Notch2 Tsc22d1 Gimap6 Serpina3g Gata1 Grk4 Pde4b Vmn1r58 Msi2 Klhl41 Tnfrsf10b Ms4a6c Ifitm1 Irf8 Fosb Itga2b Sox4 Cd34 Slamf8 Ikzf2 Smco1 Ctcfl Pan3
TD genes (up-regulated)	Ltb4r1 Lta4h Ltf Lrg1 Ly6c1 Cfp Chd7 Lcn2 Cebpe Ly6c2 Ceacam1 Chil3 Tmem38b Lbp Tmem53 Cd97 Ms4a3 Tmem2 Msrb1 Mrgpra2b Chst12 Ly6i Mt1 Cd177 Klk14 Gsr Gstm1 Mpo Gsn Ccdc125 Camp Mtus1 Tmsb4x Chst13 Tmed3 Gpx3 Mpeg1 Arsb C3 App Atp6ap2 Atp6v0c Hk3 Bzrap1 Atp8b4 Aoah Cldn15 B430306N03Rik Tifab Tmx4 Bid Gpr141 B4galnt1 Aldh3b1 Alas1 B4galt6 Anxa3 Baz1a Mogat2 Agpat2 Manf Gpc1 Afap1 Hoxd11 Clec12a Kctd20 Adssl1 Tfec Mgst2 Ncam1 Clec4a2 Mapk13 Tcn2 Hp Ethe1 Ero1l Actn1 F10 Elane Tanc1 Mgst1 Tnfsf13 Clec5a F13a1 Ncf1 Mapkapk3 Dstn Kcnip3 F630028O10Rik Hsd11b1 Syngr2 Csf2ra Abca13 Mgl2 Dok3
Gran-state IH genes	Plod3 Rhox8 G6pdx Nhsl2 Tuba8 Rasgrp2 Slc28a2 Soat1 Prdx5 Rrm2 Pigr Surf4 Tyrobp Rps6ka1 Cyba Igsf6 Pgd
Gran-state MS genes	Itgb2 Sun2 Fcgr3 Ngp Rgcc Pygl 4632428N05Rik Slpi Pilra Glrx Papss2 Nrg1 Syne1 Siglece Stom Dgat2 Phkb Ncf4 Med21 Pnkp Plin3 Rps6ka2 Gatm Rab32 Ctsg Id2 Gca 1700020L24Rik Tshr Trem3 Mefv Prss57 Pglyrp1 Gfi1 Fgl2 Gas7 Ptgr1 Itgam Fam49b Prtn3 Svip Rhou Neu1 4933440M02Rik Unc93b1 Mcemp1 Mcu S100a8 Pxylp1 Nucb2 Far2 Pde2a Ptgs1 AB124611 Sema4a Dmkn S100a9 Hvcn1 Gda G0s2 Slc40a1 Pram1 Prkcb Galns Pilrb2 Trim45 Fam160a2 Tom1 Fam101b Olr1 Mgam Pilrb1 Cybb Vcam1 Sgms2 Prom1 Fcnb 1100001G20Rik

Supplementary Table 5: (Supplementary to Supplementary Fig. 11 and Figure 5c of main text.) The list of MS, IH and TD genes in Multi-lineage to Gran transition of myelopoiesis data, with the orders of appearance corresponding to the positions from top to bottom in the figure.The genes marked with red color are top genes used to calculate gene expression trend in Figure 5c of main text.

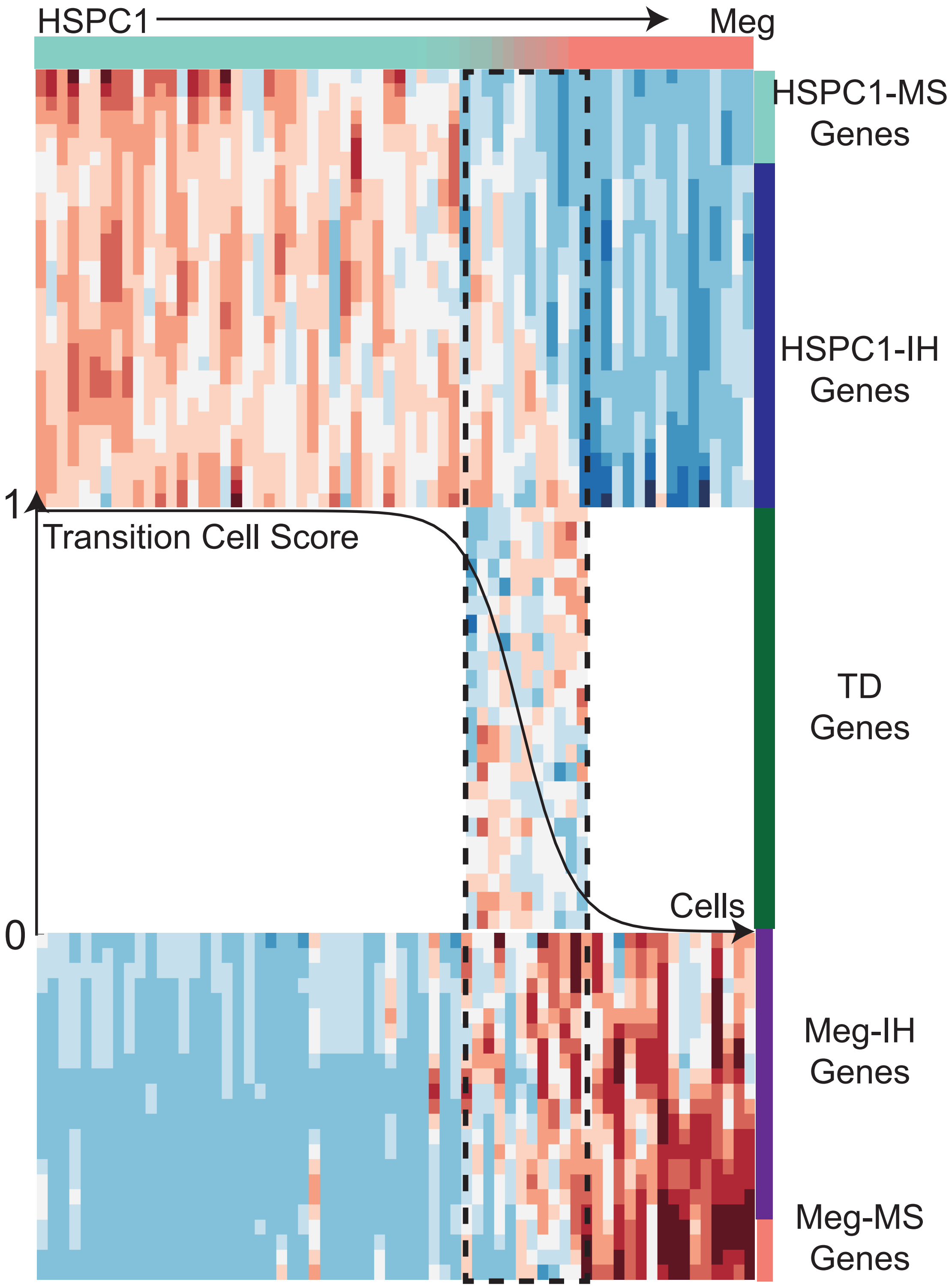

Supplementary Fig. 12: Transcendental results of myelopoiesis data for HSPC to Meg cell transitions.

Genes Group	Genes Name
HSPC-state MS genes	Mn1 Mgst1 Prtn3 Pan3 Flt3 Lbp Ifitm1
HSPC-state IH genes	Mgst2 Gfi1 Gstm1 Gvin1 Kdm6b Selplg H2-Q4 Tcf7l2 Wfdc17 Gm8979 Ctss Gimap1 Gm4070 BC035044 Dntt Ikzf2 Mecom Sox4 Notch2 Cd34 Gpc1 Zfp608 Ctcfl Gcnt2 Arglu1
TD genes (up-regulated)	Nrgn Smim5 Slpi Pbx1 Slc22a3 Tmsb4x Angpt1 Slc14a1 Ms4a3 Gata1 Arrb2 Pdia6
TD genes (down-regulated)	Hlf Gimap6 Gm1966 Emb Dusp2 Gm17821 Rtp4 Samsn1 Eltd1 Gm16287 Gm17757
Meg-state IH genes	Fyb Elane Ccr2 Zfpm1 Gp1ba Vezf1 Gfi1b Icam4 Treml1 Car1 Ctsg F13a1 F2rl2 Cst7 Pf4 F2r Itga2b Mfsd2b Rab27b
Meg-state MS genes	Ly6c2 Tuba8 Sdpr Sdf2l1

Supplementary Table 6: (Supplementary to Supplementary Fig. 12.) The list of MS, IH and TD genes in HSPC to Meg transition of myelopoiesis data, with the orders of appearance corresponding to the positions from top to bottom in the figure.

The Lymphoid Lineage Differentiation Dataset

The lymphoid lineage differentiation dataset analyzed in main text is from GSE100037 with cells sequenced by CEL-Seq 2 platform. For quality control, we kept 2,018 cells with more than 200 genes expressed and 2000 total UMI counts. Prior to MuTrans analysis, we selected 2000 highly variable genes as the input using vst method in Seurat pipeline. The processed input gene expression matrix and script to reproduce all the supplementary results for EMT data is available at `https://github.com/cliffzhou92/MuTrans-release`.

In Supplementary Fig. 13 we showed the supplementary figures in MuTrans analysis of the dataset. Of note, from the two-dimension embedding and the dynamical manifold, we observed that two streams of transition cells can become pDCs – one stream from lymphoid progenitors and one from the multi-potent progenitor cells that can also give rise to B cells. For the Pre-B cells, we also witnessed three sources of transition cells: from Pro-B cells, lymphoid progenitors or multi-potent progenitor cells shared with pDCs. We listed the gene analysis result in the Pro-B to Pre-B transition in Supplementary Table 7.

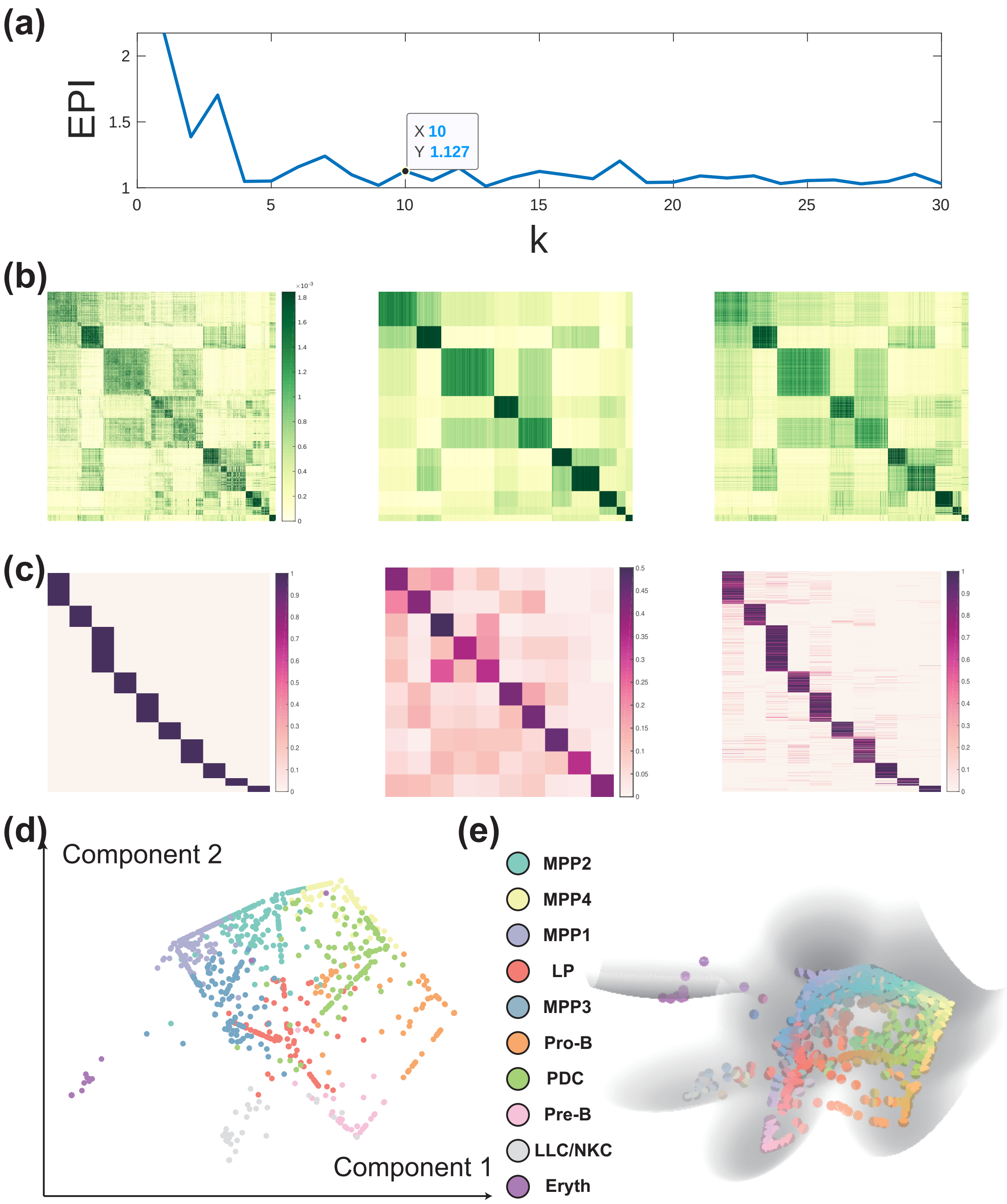

Supplementary Fig. 13: MuTrans results for the lymphoid lineage differentiation data. (a) Sorted eigenvalues of cell-cell resolution rwTPM.(b)The rwTPMs constructed from cell-cell (left), cluster-cluster (middle) and cell-cluster (right) resolutions. Note that while cluster-cluster resolution keeps the main block structure of original cell-cell resolution rwTPM, the cell-cluster resolution TPM recovers finer details. (c) The obtained cell-cluster assignment (left), cluster-cluster transition probability matrix (middle) and cell-cluster membership matrix (right) through optimization, which jointly induce the optimal rwTPMs at cluster-cluster and cell-cluster resolution in (a). (d) The constructed transition coordinates ξ as the input to dynamical manifold, based on averaging the two-dimensional embedding in (a) by membership matrix . (e) The constructed dynamical manifold with cells colored by their membership in different clusters.

Genes Group	Genes Name
Pro-B-state MS genes	Gm37324 RP23-341H2 Gm43238 Gm44443 Fcrla Cacna1e Bach2 Hvcn1 Gm17555 Gm43294 Gm10719 Ikzf3 Gm42870 Gm26905 Gm10715 Gm10717 Gm21833 Gm13936 Gm31258 Gm21962 Gm17615 Gm29055 Igkc Gm37158 Gm21738 Gm37746 F830016B08Rik Gm37900 Filip1l A430073D23Rik Gm42445 Ms4a1 4930426D05Rik Pim2 Iglc2 Gm37954 Steap4 Gm26870 Atp1b1 Fam129c Gm21750 Ciita Klf2 Gm10660 Gm44044 Scd1 Gm38142 Prkcg A530030E21Rik Ighm Trib2 Iglv1 Nfkbia Gm10800 Pou2f2 Gimap3 Gm37404 Cpm Iglc1 Cd74 Gm37951 Bhlhe41 H2-Ob Mme Cd83 Cd2 Gm42519 Gm42653 Gm37403 Gm42513 Lynx1 Rag1 Ly6d Cd79b Siglecg Gm37903 Rasgrp1 Bank1 Gm37761 Mfhas1 Relb Spib Nid1 Sertad4 AI838599 Gm43445 H2-Aa Col5a3 Adm Ahnak Gm4117 Cd55 Krt222 Cybb Gm43305 Blk Cxcr5 Fcmr H2-Eb1 Ccr7 Gimap4 Hck Ifi30
Pro-B-state IH genes	Gm37750 Arl5c Tyrobp Ublcp1 Gnas Cyp4f18 H2-DMb1 Gm37234 Dnase1l3 Rgs2 Ncf1 Dok3 Cd19 Grap BC094916 Nkain3 Slfn2 Plscr1 Edem3 Klf3 Klhl14 Gm26300 Tifa Gbp10 Gas7 Ctss Gm15987 Ltb Gm26740 Ctsh Apba2 Irf8 Chst3 Mid1 Gm44231 Ctsb Ly86 Tnfrsf13c Nfkbiz Sepp1 Adgre5 Btla Rassf4 Ser-pinb1a Xcr1 Cmah Foxo1 RP24-309B14.1 Kmo Cea-cam1 Gm44189 Cd180 Irf4 Clec2d Itgam Atp6v0a1 Psap Maf Lipc Ets1 Smtnl2 Grb7 RP24-362L9 Ighd Tmsb4x Gm9844

TD genes (up-regulated)	Depdc1a Dek Dhfr Ddx21 Dkc1 Dctpp1 Dlg2 Dbf4 Cycs Dlgap5 Cyc1 Dntt Dpy30 Drc7 Crip1 Dtl Cntln Mcm10 Mcm2 Dtymk Mbd2 Mcm3 Mb21d1 Mcm4 Mcm5 Mcm6 Mcm7 Cnn3 Mki67 Melk Mns1 Mis18bp1 Mif Mogs Mad2l1 Mrpl12 Cmc2 Mrto4 Dut Lyar Mtfr2 Clspn Dynll1 Mthfd1 Mxd3 E2f2 Cks2 Mybbp1a E2f8 Cks1b Mybl2 Lta4h Myl10 Ckap5 Ect2 Lrrc59 Nadk2 Ckap2l Nasp Lockd Ckap2 Ncapd2 Eif5a Ncapd3 Emp1 Cit Lmnb1 Ncapg Ncapg2 Eprs Ncaph Ncl Hmbs Esco2 Hjurp Hist2h2aa1 Hmgb1 Lig1 Ndc1 Hist1h4i Hmgb2 Wdhd1 Vpreb1 Vim Ybx1 Usp1 Urod Ybx3 Ung Ndc80 Hist1h2bc Ydjc Uhrf1 Hmgb3 Espl1 Ube2s Ube2c Zfp367 Ubac1 Neil3 Cep55 Hist1h2ab Zwilch Nek2 Hmgn2 Tyms Lgals1 Sapcd2 Rrm1 Etf1 Rrm2 Rps27l Nhp2 Rpa2 Ccnf Rpa1 Hmmr Ccne2 Hist1h1e Txnl1 Ccnb2 Ccne1 Ccnd3 Ccnb1 Nme1 Cenpw Set Rgcc Bcat1 Ccna2 Aurkb Bfsp2 Sgol1 Noa1 Aurka Rexo2 Tubb5 Ccdc34 Sgol2a Aunip Hist1h1b Ccdc18 Reep4 Ldha Birc5 Hnrnpab Atpif1 Nolc1 Cbx5 Shcbp1 Rcc1 Tubb4b Casc5 Nop10 Blm Atp5g3 Shmt1 Cenpp Cdc20 Bok Carnmt1 Hirip3 Bola3 Car2 Atp5g1 Cdc25c Exo1 Nop56 Brca1 Tuba4a Shmt2 Calr Hsp90aa1 Cad Brca2 Atp5b Cacybp Rangap1 Kpna2 C330027C09Rik C1qbp Bub1b Npm1 Bub1 Cdc34 Cenpn Brip1 Tuba1c Atp1b3 Ranbp1 Hells Ska1 Ezh2 Nt5dc2 Cdc45 Tuba1a Hspd1 Cenpm Slbp

Pre-B-state IH genes	Stag3 Odc1 Knstrn Ighg2b Tomm40 Cenpa Hdgf Gm4950 Psmb3 Ppat Tomm5 Oat Ass1 Ssx2ip Igll1 Arntl Acsl5 Prdx6 Cdt1 Idh3a Pmf1 Psrc1 Gm11273 Glo1 Tmem14c Anxa1 Pde2a Fabp5 Gpsm2 Cdc6 Prdx2 Pnp2 Nucks1 Ap3s1 H1fx Slc16a1 Srm Tifab Hspe1 Ppia Gm10053 Fen1 Tmem97 Pitrm1 Kif2c Alpl Fkbp4 Slc43a1 Polr2f Ifrd2 Slc43a3 Fam83d 5730488B01Rik Ankle1 Cdca7 Gnl3 Fam111a Il7r Trip13 Adgrg3 Cdkn3 Cenpe Glrx5 Ppm1g Tmem119 Tpi1 Gm6793 Phb2 3110082I17Rik Nudcd2 Gclm Slc20a1 Adk Iqgap3 Tpm4 Pgam1 Pa2g4 H2afz Trim59 Foxm1 Slc25a5 Kntc1 Ak2 Gm8225 Cdca3 Orc6 Rad51ap1 Plk4 AI506816 Anp32e Pgp Cdkn2d Pnp Psat1
Pre-B-state MS genes	Cdca8 Gm5611 Tipin Prkar2b Prr11 Atad5 Arhgef39 Kif4 Arl6ip1 Cdk6 Fut8 Rad51 Pcna Arhgap19 Gm10184 Gmnn Arhgap11a Gsr Spc24 Tpx2 H2afx Cdkn1a Nuf2 Tcf19 Pola1 Gas2l3 Ran Clgn Gins2 Gspt1 Suv39h1 Gfra2 Atic Plk1 Racgap1 Stmn1 Kif20b Atad2 H2afy Pole Pold2 Pcna-ps2 Tacc3 Pif1 Kif15 Cenpi Kif14 Cdca5 Aspm 2810417H13Rik Tk1 Cenpk Cenpf Kif11 Incenp Smc4 Nup85 Prc1 Gm26917 Kif22 Kif18a Cenpl Stil Asf1b Kif18b Rad18 Smc2 G2e3 Cenph Nusap1 Smtn Spag5 Troap Kif23 Fam64a Anln Cdca2 Pbk Top2a Cdk1 Cdkn2c Ttk Fbxo5 Spc25

Supplementary Table 7: (Supplementary to Figure 5d of main text) The list of MS, IH and TD genes in Pro-B to Pre-B transition of lymphoid lineage data.The genes marked with red color are top genes used to calculate gene expression trend in Figure 5d of main text.

The Human HSC Differentiation Dataset

The human HSC differentiation dataset analyzed in main text is from the link `https://github.com/dpeerlab/Palantir/blob/master/data/marrow_sample_scseq_counts.csv.gz` with 4,118 cells sequenced by 10X platform. Prior to MuTrans analysis, we selected 2000 highly variable genes as the input using vst method in Seurat pipeline. The processed input gene expression matrix after filtering and script to reproduce all the supplementary results for HSC data is available at `https://github.com/cliffzhou92/MuTrans-release`. In Supplementary Fig. 14 we showed the supplementary figures in MuTrans analysis of the dataset. We also include the usage of python package of MuTrans in another example Jupyter notebook `https://github.com/cliffzhou92/MuTrans-release/blob/main/Example/example_bone_marrow-new.ipynb`, especially on the DECLARE pre-processing module to speedup the analysis of large-scale data. For this data, the tSNE dimension reduction plot was computed with 20 principle components in Scanpy.

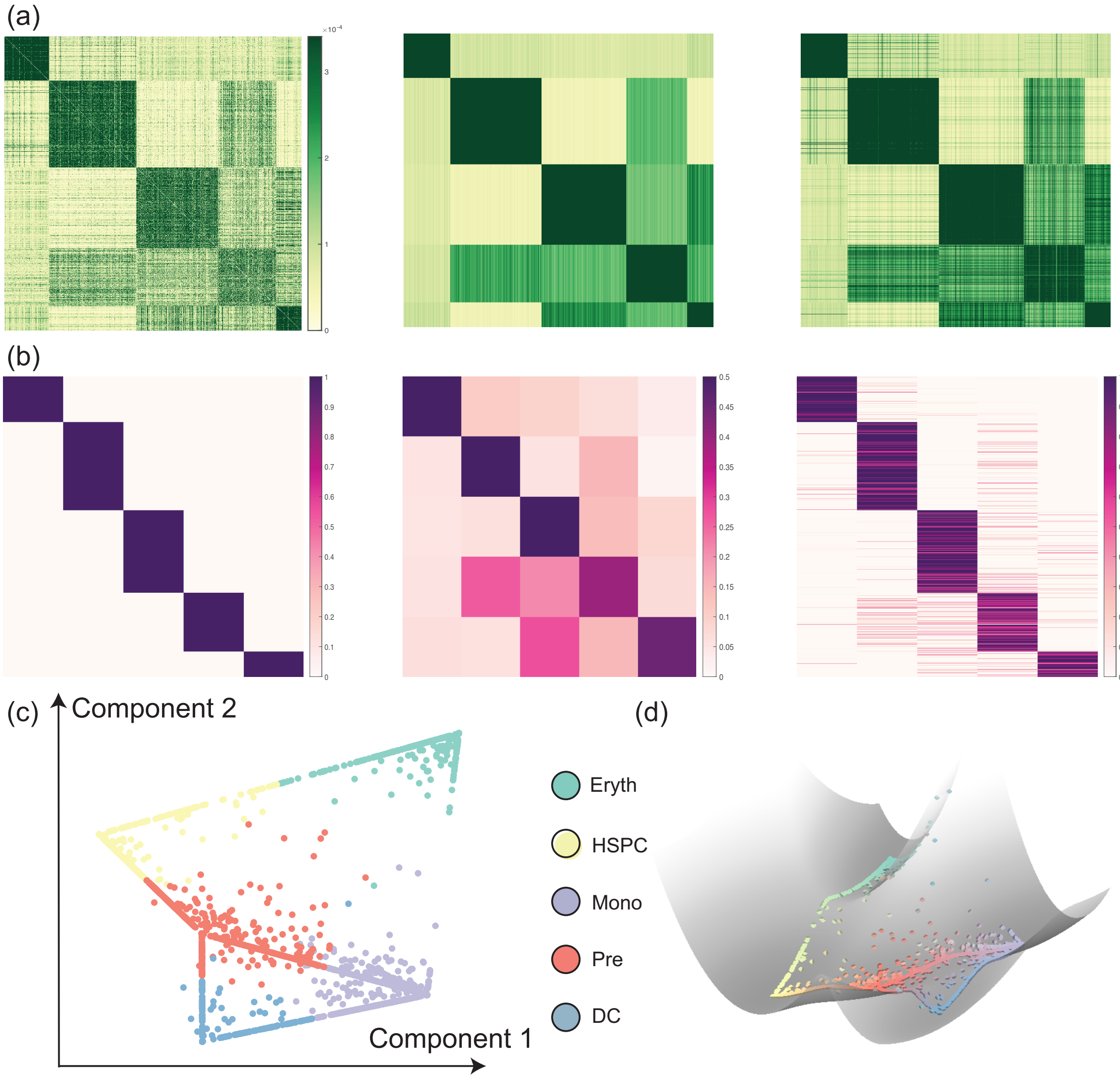

Supplementary Fig. 14: MuTrans results for the human HSC differentiation data.(a)The rwTPMs constructed from cell-cell (left), cluster-cluster (middle) and cell-cluster (right) resolutions. Note that while cluster-cluster resolution keeps the main block structure of original cell-cell resolution rwTPM, the cell-cluster resolution TPM recovers finer details. (b) The obtained cell-cluster assignment (left), cluster-cluster transition probability matrix (middle) and cell-cluster membership matrix (right) through optimization, which jointly induce the optimal rwTPMs at cluster-cluster and cell-cluster resolution in (a). (c) The constructed transition coordinates ξ as the input to dynamical manifold, based on averaging the two-dimensional embedding in (a) by membership matrix. (d) The constructed dynamical manifold with cells colored by their membership in different clusters.

The Blood Differentiation Dataset in Mouse Gastrulation

The dataset analyzed in main text is from the github repository `https://github.com/MarioniLab/EmbryoTimecourse2018`. The input expression matrix to MuTrans contains 15,875 single cells sequenced by 10X and the first 50 PCs in the blood system development. The jupyter notebook to reproduce the figures in main text is available at `https://github.com/cliffzhou92/MuTrans-release`. For this dataset, the UMAP was plotted by setting 10 neighbors with Scanpy.

Supplementary Note 4: Methods Comparison Details

MuTrans provides the functions to identify and characterize transition cells from single-cell transcriptome data, and also resolves the progressing of cell-fate transitions in complex lineages. Here we compare MuTrans with other existing approaches (such as pseudotime ordering and cell-fate bias probability) for the detection of transition cells, and capacity to unravel complex cell lineages during differentiation.

Scrutinizing Bifurcation Dynamics in iPSC Dataset

As shown in Supplementary Fig. 15a, in this dataset, MuTrans identified the cell-fate bifurcation dynamics toward En and M state (collected at day3) via two precursor states (denoted as Pre-En and Pre-M, collected at day 2 and 2.5), which are associated with streams of transition cells, as well as a minor early bifurcation into ectoderm. The transition process on dynamical manifold agrees with the trend of experimental collection time. The results are consistent with the iPSC differentiation process revealed by original paper.

We applied the existing lineage inference algorithms with the parameters and results described below.

- For Monocle 3, all the parameters are set as default. The method does not identify the cell-fate bifurcation dynamics into M and En (Supplementary Fig. 15b), and ordering M cells prior to the En cells linearly.
- For DPT, the neighbor parameter is set as 50. The method does not link the precursor states to mature states in the low dimension manifold (Supplementary Fig. 15c), and the computed pseudotime of En is also large than M.
- For PAGA, the graph layout is based on the refinement of diffusion map with max iteration number 50. The method infers the bifurcation lineage, suggesting the potential transitions between precursor and mature states, or even between the mature En and M populations (Supplementary Fig. 15d).
- For RaceID3 and StemID2, the parameters in clustering are set as default and in lineage tree inference and projection, the number of randomization for cell positions is set as 500 and p-value cut-off for link is set as 0.1. The projected lineage tree generated by RaceID3 and StemID2 implies the transition cells between precursor

and mature states, however the spanning tree does not show the bifurcation lineage (Supplementary Fig. 15e).

- For VarID, the number of principle components used is 50, the number of nearest neighbor is set as 50 and the weight alpha value is set as 0.1. All other parameters are set as default. The cluster-cluster transition probability indicated by VarID resolves the bifurcation dynamics (Supplementary Fig. 15f).

- For FateID, the clustering is based on VarID results and the targeting fates are set as En and M respectively. All other parameters are set as default. As shown in Supplementary Fig. 15g , FateID seems not produce the expected differentiation routes.

- For Palantir, the number of diffusion component used is 20 and the number of waypoints is set as 100. It does not detect branches in this dataset, while its low-dimensional manifold indeed indicates the bifurcation dynamics. Palantir manifold suggests the continuous transition from PS to precursor states.

- For PBA, the two sink cell fates are picked randomly in M or En states respectively. To compute mean first passage time (mfpt), we select the initial cell randomly from epiblast state. Due to lack of prior knowledge, all the cell growth rates are set as zero. All other parameters are set as default. The cell-fate probabilities on individual cell level can capture transition cells from Pre-M or Pre-En states to M or En states, while the distinctions of fates between Pre-En and Pre-M attractors are not very obvious. The mean first passage time (MFPT) agrees with overall trend of true collection time.

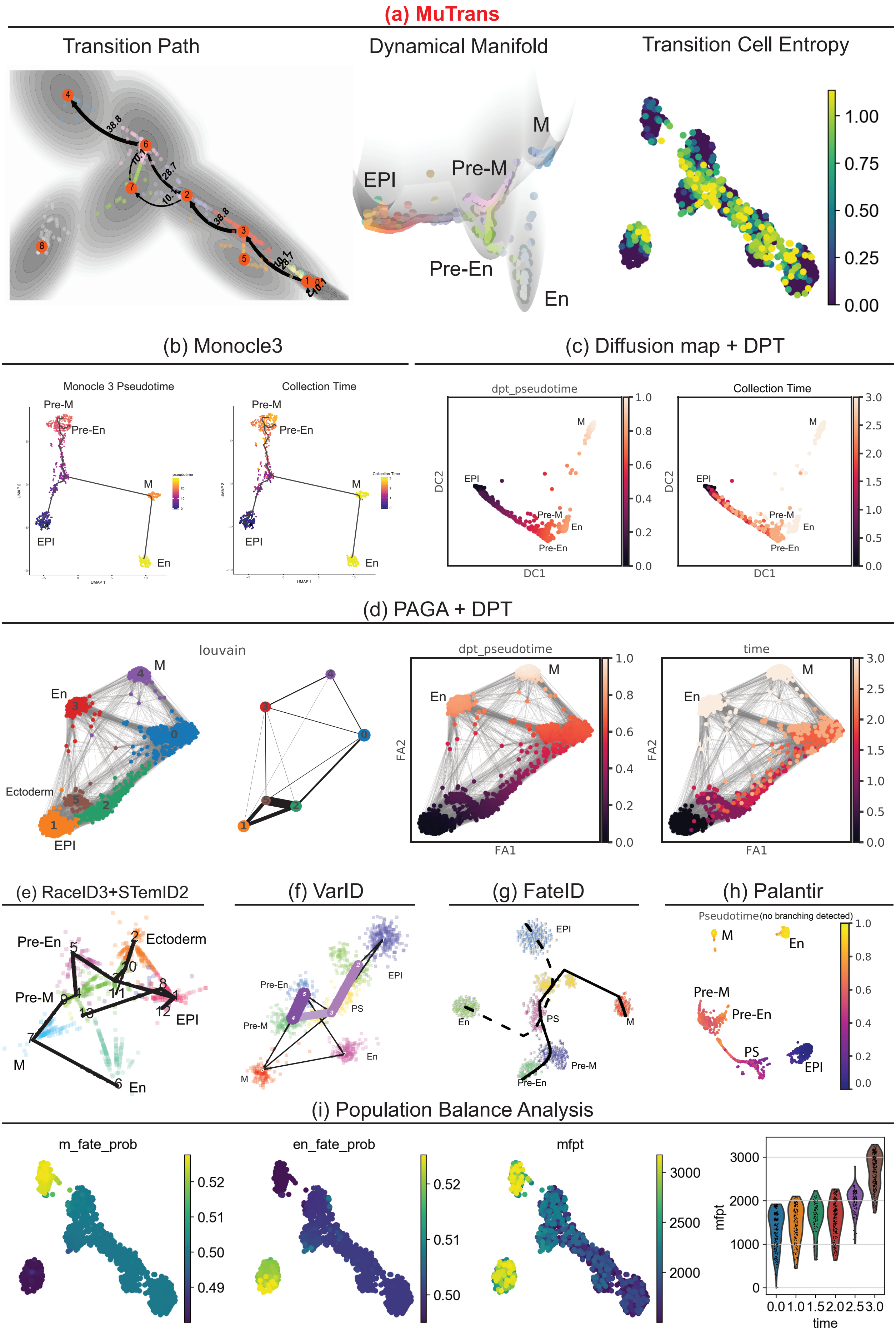
(a) MuTrans
Transition Path
Dynamical Manifold
Transition Cell Entropy
EPI
Pre-M
M
Pre-En
En
1.00
0.75
0.50
0.25
0.00
(b) Monocle3
(c) Diffusion map + DPT
Monocle 3 Pseudotime
Collection Time
dpt_pseudotime
DC1
DC2
(d) PAGA + DPT
louvain
Ectoderm
time
FA1
FA2
(e) RaceID3+STemID2
(f) VarID
(g) FateID
(h) Palantir
Pseudotime(no branching detected)
PS
(i) Population Balance Analysis
m_fate_prob
en_fate_prob
mfpt
time

Supplementary Fig. 15: Comparison of MuTrans with other lineage inference methods in iPSC data. All methods take the same pre-processed data matrix as input. (a) The most probable path tree (MPPT,left), dynamical manifold colored with MuTrans clusters (middle) and experimental collection time (right). (b) Lineage inference and pseudotime ordering (left) by Monocle 3, shown on the low dimensional reduction of umap. (c) Lineage inference and pseudotime ordering by diffusion pseudotime (DPT), shown on the low dimensional reduction of diffusion map. (d) Graph representation of cells and clusters (left) and DPT (right) by PAGA. The graph representation is based on the refinement of diffusion map, and clustering is done by Louvain method. (e) Lineage inference and projection by RaceID3 and StemID2. The colors represents clustering results by RaceID3, and the spanning tree (black lines) and low-dimension projection are calculated by StemID2. (f) Lineage inference by the probability graph of VarID. The colors represents clustering results by VarID, and the thickness of lines represents the value of transition probability. (g) Lineage inference by FateID plotted on tSNE dimension reduction. The colors represents clustering results by VarID, and the lines represent the inferred routes targeted at En or M. (h) Low dimension manifold and pseudotime obtained from Palantir. (i) The cell fate probability toward En or M states and the mean first passage time (mfpt) from stem cells, calculated by PBA.

Dissecting Complex Lineage in Myelopoiesis Dataset

In this dataset, MuTrans revealed complex branching dynamics mediated by the hub multi-lineage basin, where multiple streams of transition cells depart (Supplementary Fig. 16a).

- For Monocle 3, all the parameters are set as default. The method seems not identify the bifurcations into Gran/Mono or Eryth/Meg lineages. Also the lineage tree suggests the transition from multi-lineage cells to Gran/Mono lineages is very sharp, with hardly any transition cells exists (Supplementary Fig. 16b).
- For DPT, the neighbor parameter is set as 10. The low dimensional manifold or pseudo time does not resolve the bifurcations (Supplementary Fig. 16c).
- For PAGA, the graph layout is based on the refinement of diffusion map with max iteration number 50. The method highlights multi-lineage state as the hub in cell lineage and resolves the overall bifurcations (Supplementary Fig. 16d).
- For RaceID3 and StemID2, the parameters in clustering are set as default and in lineage tree inference and projection, the number of randomization for cell positions is set as 100 and p-value cut-off for link is set as 0.1. The projected lineage tree suggests transition cells exist between multi-lineage cells and other cell states (Supplementary Fig. 16e).
- For VarID, the number of principle components used is 100, the number of nearest neighbor is set as 10 and the weight alpha value is set as 10. VarID can depict the overall cell lineage (Supplementary Fig. 16f).
- For FateID, the clustering is based on VarID results and the targeting fates are set as myeolocyte and monocyte respectively. All other parameters are set as default. FateID identifies the differentiation route toward Mono lineage (Supplementary Fig. 16g).
- For Palantir, the number of diffusion component used is 10 and the number of waypoints is set as 70. It does not detect branches in this dataset by default setting, and its low-dimensional manifold separates multi-lineage cells into two disconnected regions (Supplementary Fig. 16h).

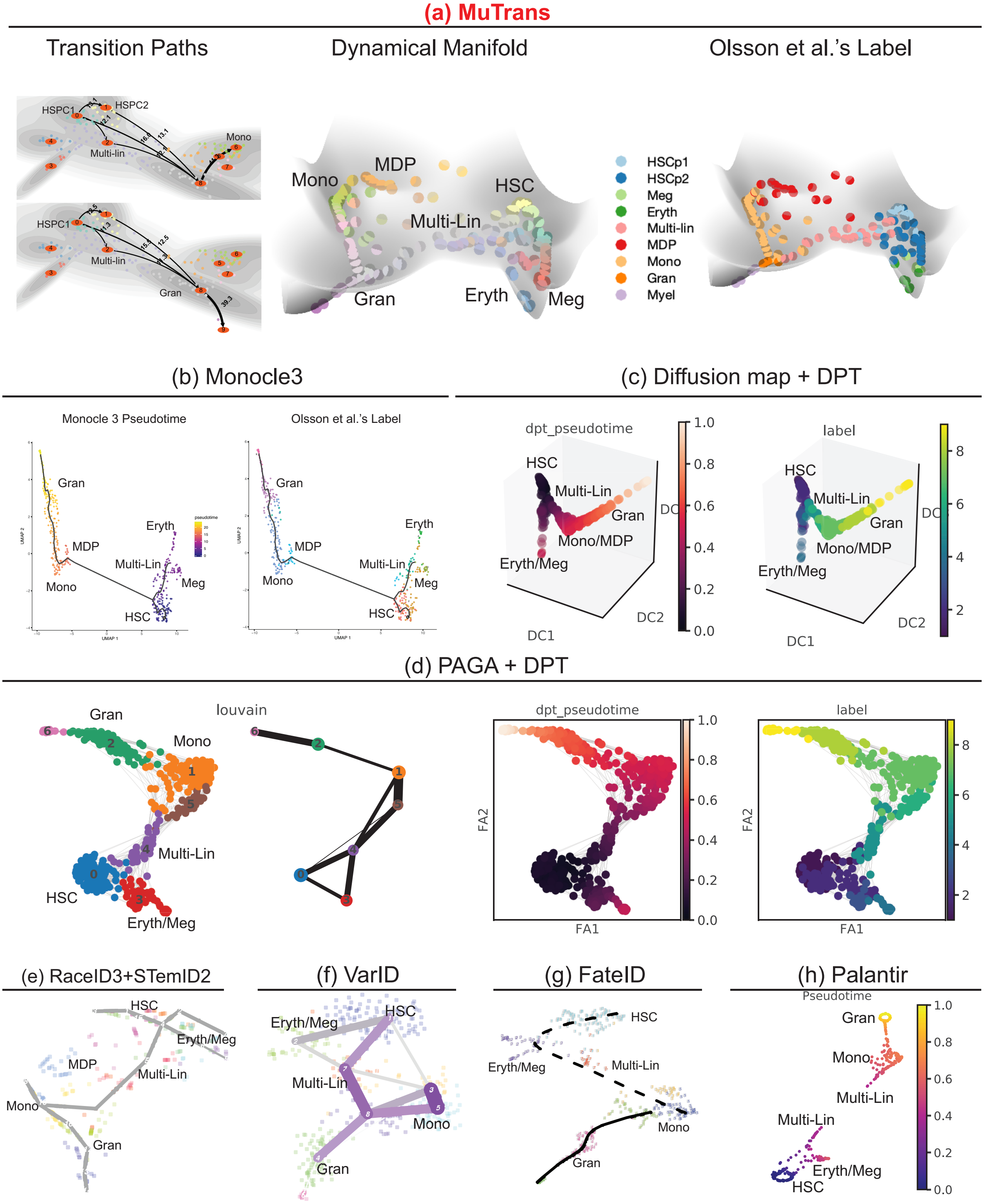

(a) MuTrans
Transition Paths
Dynamical Manifold
Olsson et al.'s Label
HSPC1
HSPC2
Multi-lin
Mono
Gran
MDP
Mono
HSC
Multi-Lin
Gran
Eryth
Meg
HSCp1
HSCp2
Meg
Eryth
Multi-lin
MDP
Mono
Gran
Myel
(b) Monocle3
Monocle 3 Pseudotime
Olsson et al.'s Label
Gran
MDP
Mono
Eryth
Multi-Lin
Meg
HSC
(c) Diffusion map + DPT
dpt_pseudotime
label
HSC
Multi-Lin
Gran
Mono/MDP
Eryth/Meg
DC1
DC2
(d) PAGA + DPT
louvain
Gran
Mono
Multi-Lin
HSC
Eryth/Meg
dpt_pseudotime
label
FA1
FA2
(e) RaceID3+STemID2
HSC
Eryth/Meg
MDP
Multi-Lin
Mono
Gran
(f) VarID
Eryth/Meg
HSC
Multi-Lin
Mono
Gran
(g) FateID
HSC
Eryth/Meg
Multi-Lin
Mono
Gran
(h) Palantir
Pseudotime
Gran
Mono
Multi-Lin
Multi-Lin
Eryth/Meg
HSC

Supplementary Fig. 16: Comparison of MuTrans with other lineage inference methods in myelopoiesis data. All methods take the same pre-processed data matrix as input. (a) The most probable path (left), dynamical manifold colored with MuTrans clusters (middle) and labels in original publication (right). (b) Lineage inference and pseudotime ordering (left) by Monocle 3, shown on the low dimensional reduction of umap. (c) Lineage inference and pseudotime ordering by diffusion pseudotime (DPT), shown on the low dimensional reduction of diffusion map. (d) Graph representation of cells and clusters (left) and DPT (right) by PAGA. The graph representation is based on the refinement of diffusion map, and clustering is done by Louvain method. (e) Lineage inference and projection by RaceID3 and StemID2. The colors represents clustering results by RaceID3, and the spanning tree (black lines) and low-dimension projection are calculated by StemID2. (f) Lineage inference by the probability graph of VarID. The colors represents clustering results by VarID, and the thickness of lines represents the value of transition probability. (g) Lineage inference by FateID plotted on tSNE dimension reduction. The colors represents clustering results by VarID, and the lines represent the inferred routes targeted at Gran or Mono. (h) Low dimension manifold and pseudotime obtained from Palantir.

Probing Transition Cells in Transitions toward En/M States of iPSC Dataset

To show the capability of MuTrans to quantitatively distinguish between stable and transition cells, in Supplementary Fig. 17 we compared with other ways to identify transition cells in iPSC dataset based pseudotime or cell fate probability. In both Pre-M to M and Pre-En to En transitions, MuTrans identify the transition cells that are well-characterized by the expression dynamics of TD, IH and MS genes (Figure 4 in main text and Supplementary Table 4). Here DPT and Monocle 3 pseudotime during the processes witness the sharp switch-like transitions, resulting in very few transition cells to be detected between attractors. We also observed that the duration of pseudotime spent in stable cells is significantly shorter than that in transition cells (indicated by the Y-label in Supplementary Fig. 17). This is because DPT and Monocle 3 pseudotime is defined based on manifold distance in low dimensional space instead of cell transition dynamics. In low dimensional space, the stable cells are crowded together, while the transition cells are on the paths connecting different clusters of cells. Thus the manifold distances within stable cells are shorter than the distance along the transition path. Hence, wide-applied pseudotime might not serve as the natural tool to inspect transition cells in this dataset. We also compared the ordering of cells produced by Fate ID cell fate probability and Palantir pseudo time. We found that the two measures change continuously during the transition, lacking resolutions to distinguish between stable and transition cells in the interested cell-fate switch.

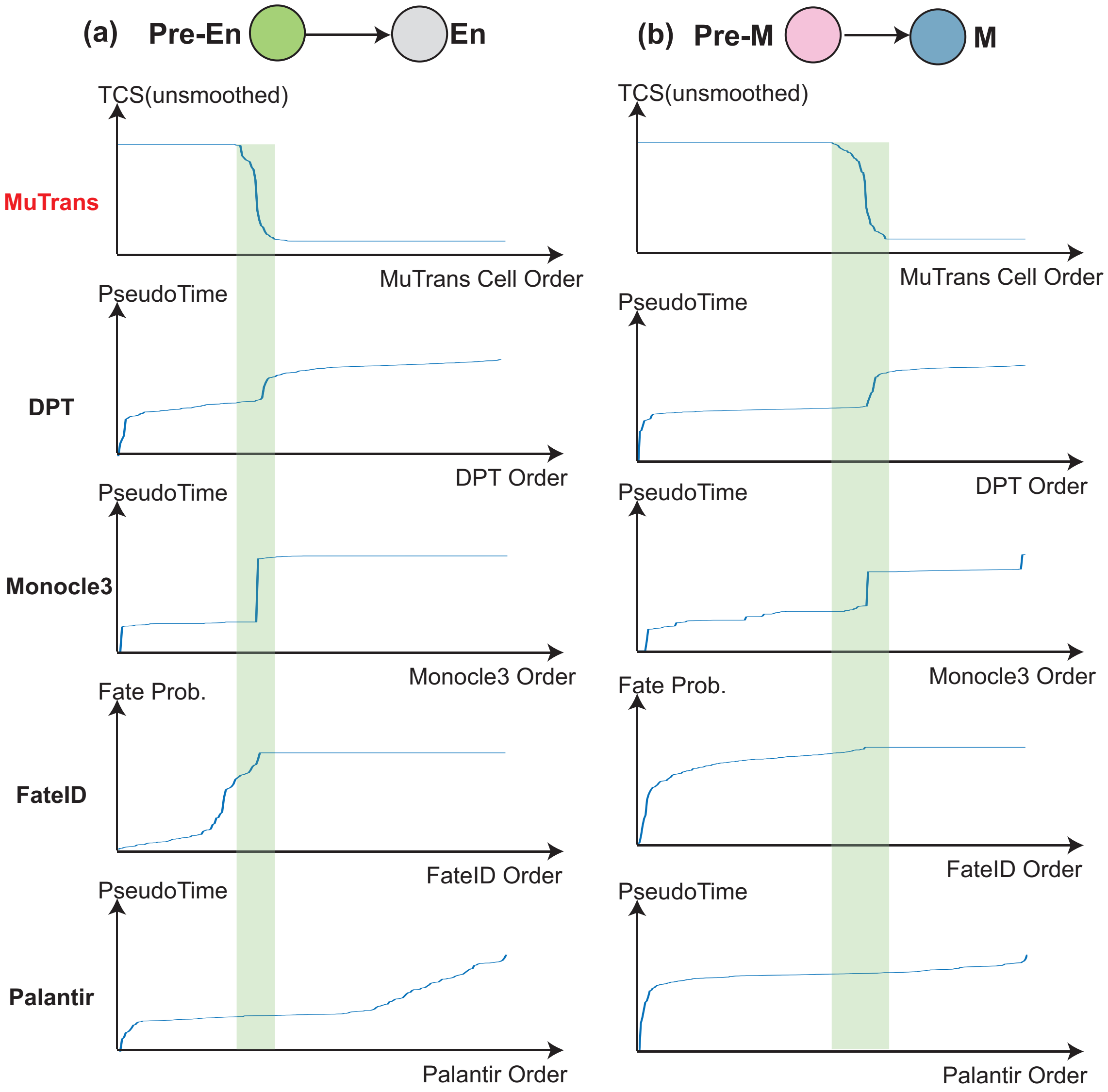

Supplementary Fig. 17: Comparison of MuTrans transition cell score (TCS), Monocle 3 pseudotime, diffusion pseudotime(DPT), FateID fate bias probability and Palantir pseudotime (from top to bottom) to detect transition cells in (a) Pre-En to En and (b) Pre-M to M transition processes of iPSC data. The y-axis denotes the different measures and the x-axis represents the cells ordered by the corresponding measures.Green box indicates the position of MuTrans identified transition cells.

MuTrans as a Pseudotime Method

The main functions of MuTrans focus on probing the transition cells between stable states instead of calculating the pseudotime. However, based on the transition cell analysis of MuTrans, we can also naturally define a uniform pseudotime ordering,

$$\mathcal{PT}(x) = \sum_{k=1}^{K} \rho_k(x)\mathcal{L}_k,$$

where L_k is the distance between state S_k and root state in the inferred lineage. As shown in Supplementary Table 8, measured by the correlation with experimental collection time of iPSC dataset, the pseudotime ordering generated by MuTrans yields similar results with diffusion pseudotime (DPT) and has the larger correlation compared with the pseudotime of Palantir or PBA.

**Methods**	**Corr(Pearson)**	**Corr(Kendall)**	**Corr(Spearman)**
MuTrans	0.94	0.80	0.93
DPT	0.94	0.84	0.95
Palantir	0.87	0.55	0.72
PBA	0.69	0.57	0.73

Supplementary Table 8: Comparison of MuTrans with DPT and Palantir to calculate the pseudotime ordering in iPSC data.

References

[1] Hanggi P. Escape from a metastable state. Journal of Statistical Physics. 1986;42(1-2):105–148.

[2] Graf T, Enver T. Forcing cells to change lineages. Nature. 2009;462:587.

[3] Strasser M, Theis FJ, Marr C. Stability and multiattractor dynamics of a toggle switch based on a two-stage model of stochastic gene expression. Biophys J. 2012;102(1):19–29.

[4] Ge H, Qian H, Xie XS. Stochastic phenotype transition of a single cell in an intermediate region of gene state switching. Phys Rev Lett. 2015;114(7).

[5] Moris N, Pina C, Arias AM. Transition states and cell fate decisions in epigenetic landscapes. Nature Reviews Genetics. 2016;17(11):693.

[6] Brackston RD, Lakatos E, Stumpf MPH. Transition state characteristics during cell differentiation. PLoS Computational Biology. 2018;14(9):e1006405.

[7] MacLean AL, Hong T, Nie Q. Exploring intermediate cell states through the lens of single cells. Current opinion in systems biology. 2018;9:32–41.

[8] Kramers HA. Brownian motion in a field of force and the diffusion model of chemical reactions. Physica. 1940;7(4):284–304.

[9] Freidlin MI, Szücs J, Wentzell AD. Random perturbations of dynamical systems. vol. 260. Springer Science & Business Media; 2012.

[10] Marr C, Zhou JX, Huang S. Single-cell gene expression profiling and cell state dynamics: collecting data, correlating data points and connecting the dots. Current opinion in biotechnology. 2016;39:207–214.

[11] Coifman RR, Lafon S. Diffusion maps. Applied and Computational Harmonic Analysis. 2006;21(1):5–30.

[12] Haghverdi L, Buettner F, Theis FJ. Diffusion maps for high-dimensional single-cell analysis of differentiation data. Bioinformatics. 2015;31(18):2989–2998.

[13] Haghverdi L, Buettner M, Wolf FA, Buettner F, Theis FJ. Diffusion pseudotime robustly reconstructs lineage branching. Nature methods. 2016;13(10):845.

[14] E W, Li T, Vanden-Eijnden E. Applied Stochastic Analysis. vol. 199. American Mathematical Soc.; 2019.

[15] Ocone A, Haghverdi L, Mueller NS, Theis FJ. Reconstructing gene regulatory dynamics from high-dimensional single-cell snapshot data. Bioinformatics. 2015;31(12):i89–i96.

[16] Marco E, Karp RL, Guo G, Robson P, Hart AH, Trippa L, et al. Bifurcation analysis of single-cell gene expression data reveals epigenetic landscape. Proceedings of the National Academy of Sciences. 2014;111(52):E5643–E5650.

[17] Matsumoto H, Kiryu H. SCOUP: a probabilistic model based on the Ornstein–Uhlenbeck process to analyze single-cell expression data during differentiation. BMC bioinformatics. 2016;17(1):232.

[18] Buettner F, Theis FJ. A novel approach for resolving differences in single-cell gene expression patterns from zygote to blastocyst. Bioinformatics. 2012;28(18):i626–i632.

[19] Ahmed S, Rattray M, Boukouvalas A. GrandPrix: scaling up the Bayesian GPLVM for single-cell data. Bioinformatics. 2018;p. bty533–bty533.

[20] Campbell KR, Yau C. Order Under Uncertainty: Robust Differential Expression Analysis Using Probabilistic Models for Pseudotime Inference [Journal Article]. PLoS Computational Biology. 2016;12(11):e1005212.

[21] Fischer DS, Fiedler AK, Kernfeld E, Genga RMJ, Hasenauer J, Maehr R, et al. Beyond pseudotime: Following T-cell maturation in singlecell RNAseq time series [Journal Article]. bioRxiv. 2017;.

[22] Weinreb C, Wolock S, Tusi BK, Socolovsky M, Klein AM. Fundamental limits on dynamic inference from single-cell snapshots. Proceedings of the National Academy of Sciences. 2018;115(10):E2467–E2476.

[23] Zhang J, Nie Q, Zhou T. Revealing dynamic mechanisms of cell fate decisions from single-cell transcriptomic data. Frontiers in genetics. 2019;10:1280.

[24] Setty M, Kiseliovas V, Levine J, Gayoso A, Mazutis L, Pe'er D. Palantir characterizes cell fate continuities in human hematopoiesis. bioRxiv. 2018;.

[25] Peters G, Crespo F, Lingras P, Weber R. Soft clustering–fuzzy and rough approaches and their extensions and derivatives. International Journal of Approximate Reasoning. 2013;54(2):307–322.

[26] E W, Li T, Vanden-Eijnden E. Optimal partition and effective dynamics of complex networks. Proceedings of the National Academy of Sciences, USA. 2008;105(23):7907–7912.

[27] Jin S, MacLean AL, Peng T, Nie Q. scEpath: energy landscape-based inference of transition probabilities and cellular trajectories from single-cell transcriptomic data. Bioinformatics. 2018;34(12):2077–2086.

[28] Chen Z, An S, Bai X, Gong F, Ma L, Wan L. DensityPath: an algorithm to visualize and reconstruct cell state-transition path on density landscape for single-cell RNA sequencing data. Bioinformatics. 2018;.

[29] E W, Vanden-Eijnden E. Transition-path theory and path-finding algorithms for the study of rare events. Annual Review of Physical Chemistry. 2010;61:391–420.

[30] E W, Vanden-Eijnden E. Towards a theory of transition paths. Journal of statistical physics. 2006;123(3):503.

[31] Metzner P, Schütte C, Vanden-Eijnden E. Transition path theory for Markov jump processes. Multiscale Modeling and Simulation. 2009;7(3):1192–1219.

[32] Noé F, Schütte C, Vanden-Eijnden E, Reich L, Weikl TR. Constructing the equilibrium ensemble of folding pathways from short off-equilibrium simulations. Proceedings of the National Academy of Sciences. 2009;106(45):19011–19016.

[33] Bowman GR, Pande VS, Noé F. An introduction to Markov state models and their application to long timescale molecular simulation. vol. 797. Springer Science & Business Media; 2013.

[34] Li T, Shi J, Wu Y, Zhou P. On the Mathematics of RNA Velocity I: Theoretical Analysis. CSIAM Transactions on Applied Mathematics. 2021;2(1):1–55.

[35] Scherer MK, Trendelkamp-Schroer B, Paul F, Prez-Hernndez G, Hoffmann M, Plattner N, et al. PyEMMA 2: A Software Package for Estimation, Validation, and Analysis of Markov Models. Journal of Chemical Theory and Computation. 2015 Oct;11:5525–5542.

[36] Fischer DS, Fiedler AK, Kernfeld EM, Genga RM, Bastidas-Ponce A, Bakhti M, et al. Inferring population dynamics from single-cell RNA-sequencing time series data. Nature biotechnology. 2019;37(4):461.

[37] Metzner P, Schütte C, Vanden-Eijnden E. Illustration of transition path theory on a collection of simple examples. The Journal of Chemical Physics. 2006;125(8):084110.

[38] Pastushenko I, Brisebarre A, Sifrim A, Fioramonti M, Revenco T, Boumahdi S, et al. Identification of the tumour transition states occurring during EMT. Nature. 2018;556(7702):463.

[39] Olsson A, Venkatasubramanian M, Chaudhri VK, Aronow BJ, Salomonis N, Singh H, et al. Single-cell analysis of mixed-lineage states leading to a binary cell fate choice. Nature. 2016;537(7622):698.
