# Supplementary material for: Dissecting transition cells from single-cell transcriptome data through multiscale stochastic dynamics
Source: Nat Commun. 2021 Sep 23;12:5609. doi: 10.1038/s41467-021-25548-w (PMC8460805; doi:10.1038/s41467-021-25548-w)
Supplement: Supplementary file 3 — Reporting Summary [file 41467_2021_25548_MOESM3_ESM.pdf]

nature portfolio

Corresponding author(s): Tiejun Li, Qing Nie

Last updated by author(s): Aug 3, 2021

Reporting Summary

Nature Portfolio wishes to improve the reproducibility of the work that we publish. This form provides structure for consistency and transparency in reporting. For further information on Nature Portfolio policies, see our Editorial Policies and the Editorial Policy Checklist.

Statistics

For all statistical analyses, confirm that the following items are present in the figure legend, table legend, main text, or Methods section.

n/a	Confirmed	
☐	☒	The exact sample size (*n*) for each experimental group/condition, given as a discrete number and unit of measurement
☒	☐	A statement on whether measurements were taken from distinct samples or whether the same sample was measured repeatedly
☒	☐	The statistical test(s) used AND whether they are one- or two-sided<br>*Only common tests should be described solely by name; describe more complex techniques in the Methods section.*
☒	☐	A description of all covariates tested
☒	☐	A description of any assumptions or corrections, such as tests of normality and adjustment for multiple comparisons
☐	☒	A full description of the statistical parameters including central tendency (e.g. means) or other basic estimates (e.g. regression coefficient) AND variation (e.g. standard deviation) or associated estimates of uncertainty (e.g. confidence intervals)
☒	☐	For null hypothesis testing, the test statistic (e.g. *F*, *t*, *r*) with confidence intervals, effect sizes, degrees of freedom and *P* value noted<br>*Give P values as exact values whenever suitable.*
☒	☐	For Bayesian analysis, information on the choice of priors and Markov chain Monte Carlo settings
☒	☐	For hierarchical and complex designs, identification of the appropriate level for tests and full reporting of outcomes
☐	☒	Estimates of effect sizes (e.g. Cohen's *d*, Pearson's *r*), indicating how they were calculated

Our web collection on statistics for biologists contains articles on many of the points above.

Software and code

Policy information about availability of computer code

Data collection	No software was used for data collection.
Data analysis	We performed the data analysis with newly developed package MuTrans in this study which is available at https://github.com/cliffzhou92/MuTrans-release. MuTrans was developed with software Matlab 2019b and platform Anaconda 4.9.2 with Python 3.7. It has the package dependencies on PyEmma 2.5.6 for transition paths analysis, and compatible with the single-cell analysis package Scanpy 1.6.0. For single-cell data analysis, we also used the package Seurat (Version 3.0) in R 3.6.

For manuscripts utilizing custom algorithms or software that are central to the research but not yet described in published literature, software must be made available to editors and reviewers. We strongly encourage code deposition in a community repository (e.g. GitHub). See the Nature Portfolio guidelines for submitting code & software for further information.

Data

Policy information about availability of data

All manuscripts must include a data availability statement. This statement should provide the following information, where applicable:

- Accession codes, unique identifiers, or web links for publicly available datasets
- A description of any restrictions on data availability
- For clinical datasets or third party data, please ensure that the statement adheres to our policy

All the datasets used in this paper are publicly available. The mouse cancer EMT data (Smart-Seq2) used in this study was downloaded from the Gene Expression Omnibus (GEO) with accession number GSE110357 (https://www.ncbi.nlm.nih.gov/geo/query/acc.cgi?acc=GSE110357). The mouse myelopoiesis data (Fluidigm C1) used in this study was downloaded from the Gene Expression Omnibus (GEO) with accession number GSE7024 (https://www.ncbi.nlm.nih.gov/geo/query/acc.cgi?acc=GSE7024). The mouse hematopoietic progenitors data (Cel-Seq2) used in this study was downloaded from the Gene Expression Omnibus (GEO) with accession

number GSE100037 (https://www.ncbi.nlm.nih.gov/geo/query/acc.cgi?acc=GSE100037). The processed human hematopoietic progenitors data (10X Chromium) used in this study was downloaded from https://github.com/dpeerlab/Palantir/blob/master/data/marrow_sample_scseq_counts.csv.gz and processed blood differentiation data (10X Chromium) in mouse gastrulation used in this study was downloaded from https://github.com/MarioniLab/EmbryoTimecourse2018. The iPSC differentiation data (single-cell RT-qPCR) used in this study was downloaded from https://www.pnas.org/highwire/filestream/29285/field_highwire_adjunct_files/1/pnas.1621412114.sd02.xlsx. The codes and trajectories for simulation data, the processed single-cell data expression matrix, the MuTrans package and scripts to reproduce the figures and results in main text and repeat the detailed analysis in SI are also available at Github (https://github.com/cliffzhou92/MuTrans-release).

Field-specific reporting

Please select the one below that is the best fit for your research. If you are not sure, read the appropriate sections before making your selection.

- [x] Life sciences
- [ ] Behavioural & social sciences
- [ ] Ecological, evolutionary & environmental sciences

For a reference copy of the document with all sections, see nature.com/documents/nr-reporting-summary-flat.pdf

Life sciences study design

All studies must disclose on these points even when the disclosure is negative.

Sample size	No biological experiment was conducted in this study and no sample-size computation was performed. We used six published single-cell datasets to demonstrate the functions and usages of our newly developed algorithm MuTrans. The datasets represented different biological systems ranging from cancer EMT, iPSC differentiation to blood differentiation, and were generated by different sequencing platforms. Therefore, it is sufficient to show the ability of MuTrans to dissect cell-fate transitions from single-cell transcriptome datasets.
Data exclusions	Standard quality control procedure for the scRNA-seq data was carried out. Specifically, for the Smart-Seq 2 raw counts in EMT dataset, we followed the established pipeline in the original publication to remove cells with high ERCC counts percentage and low total counts. For the Fluidigm C1 myelopoiesis dataset, we filtered out the outliers based on the unsupervised tSNE dimension reduction. For the CEL-Seq2 lymphoid lineage differentiation dataset, we removed low-quality cells with low gene expression numbers or UMI counts following the strategy in original paper. For the other datasets, no cells were removed following the analysis by original data contributors. These details have been described in Methods and Supplementary Note 3, and we have provided the scripts for the calculation.
Replication	No biological experiment was conducted in this study. For computational tasks, we repeated the programs with different runs and the results were reproducible. We uploaded the scripts to reproduce the results in this study to https://github.com/cliffzhou92/MuTrans-release/tree/main/Example.
Randomization	No biological experiment was conducted in this study and randomization was not relevant for computational tasks in our study.
Blinding	No biological experiment was conducted in this study and blinding was not relevant for computational tasks in our study.

Reporting for specific materials, systems and methods

We require information from authors about some types of materials, experimental systems and methods used in many studies. Here, indicate whether each material, system or method listed is relevant to your study. If you are not sure if a list item applies to your research, read the appropriate section before selecting a response.

Materials & experimental systems

n/a	Involved in the study
[x]	[ ] Antibodies
[x]	[ ] Eukaryotic cell lines
[x]	[ ] Palaeontology and archaeology
[x]	[ ] Animals and other organisms
[x]	[ ] Human research participants
[x]	[ ] Clinical data
[x]	[ ] Dual use research of concern

Methods

n/a	Involved in the study
[x]	[ ] ChIP-seq
[x]	[ ] Flow cytometry
[x]	[ ] MRI-based neuroimaging

March 2021
